# A panel of colorimetric assays to measure enzymatic activity in the base excision DNA repair pathway

**DOI:** 10.1093/nar/gkz171

**Published:** 2019-03-14

**Authors:** Eleanor Healing, Clara F Charlier, Lisiane B Meira, Ruan M Elliott

**Affiliations:** 1 Department of Nutritional Sciences, University of Surrey, Guildford, Surrey GU2 7XH, UK; 2 Department of Clinical and Experimental Medicine, University of Surrey, Guildford, Surrey GU2 7XH, UK

## Abstract

DNA repair is essential for the maintenance of genomic integrity, and evidence suggest that inter-individual variation in DNA repair efficiency may contribute to disease risk. However, robust assays suitable for quantitative determination of DNA repair capacity in large cohort and clinical trials are needed to evaluate these apparent associations fully. We describe here a set of microplate-based oligonucleotide assays for high-throughput, non-radioactive and quantitative determination of repair enzyme activity at individual steps and over multiple steps of the DNA base excision repair pathway. The assays are highly sensitive: using HepG2 nuclear extract, enzyme activities were quantifiable at concentrations of 0.0002 to 0.181 μg per reaction, depending on the enzyme being measured. Assay coefficients of variation are comparable with other microplate-based assays. The assay format requires no specialist equipment and has the potential to be extended for analysis of a wide range of DNA repair enzyme activities. As such, these assays hold considerable promise for gaining new mechanistic insights into how DNA repair is related to individual genetics, disease status or progression and other environmental factors and investigating whether DNA repair activities can be used a biomarker of disease risk.

## INTRODUCTION

There are seven major DNA repair pathways in human cells, each with capacity to repair specific lesion types, and effective functioning of these is critical for cellular survival and health (1,2). Among these, the base excision repair (BER) pathway is responsible for repairing damage caused to single bases by insults such as reactive oxygen species, ionizing radiation and alkylating agents (3). BER can also repair apurinic/apyrimidinic (AP) sites and single strand breaks (SSB) in the DNA backbone (4). If repairing a base lesion, the first step in the BER pathway is performed by a DNA glycosylase that removes the damaged base. A total of 11 DNA glycosylases have been characterized in mammalian systems to date, each specific for a particular set of the base lesions (5). Uracil DNA glycosylases (UDGs) preferentially remove uracil in DNA that has occurred by spontaneous deamination of cytosine (generating U:G mispairs) or that has been misincorporated during DNA synthesis (leading to U:A base pairs) (6,7). UDGs remove the damaged base and leave an AP site, which is a substrate for AP endonuclease (APE1). APE1 cleaves the DNA sugar-phosphate backbone at the AP site (8), leaving a one-nucleotide gap with a hydroxyl group at the free 3′ DNA strand end and a deoxyribose phosphate group (dRP) attached to the 5′ DNA strand terminus. Following APE1 incision, BER can proceed as either short-patch BER or long-patch BER. For both pathways, a polymerase acts upon the one-nucleotide gap, most commonly DNA polymerase β (POLB), adding one undamaged base in short patch BER and using its dRP lyase activity to remove the 5′ dRP from the DNA backbone at the repair site (9). Alternatively, more than one nucleotide is replaced via long-patch BER (10) with the resulting displaced DNA strand being excised by flap endonuclease (FEN1) (11). The final step in both short and long patch BER is the rejoining of the DNA backbone by either DNA ligase IIIa or DNA ligase I (12,13).

A reduction in BER activity or its dyscoordination can lead to a range of detrimental health outcomes, the most common being increased susceptibility to cancer, neurodegeneration and premature aging (14). In the general population, BER enzyme activity has been found to vary by up to 10-fold among apparently healthy individuals (15). In some cases, such variation may influence disease risk. For example, familial adenomatous polyposis colon cancer is associated with a mutation of the DNA repair enzyme MYH adenine DNA glycosylase (16), and sporadic lung cancer may be linked to reduced 8-oxoguanine DNA glycosylase (OGG1) activity (17). Conversely, an increase in BER capacity is not necessarily protective against cancer, as shown by an increase in lung cancer risk with increased alkyl adenine glycosylase (AAG) activity (18). It is clear that the association of BER capacity with health outcomes is a complex issue and one that requires further research.

While a range of methods have been used to measure DNA repair capacity, most present critical limitations such as low sensitivity, low throughput or the requirement for radio-labeling. Here, we describe the development and validation of a panel of non-radioactive assays that can determine DNA repair activities within the BER pathway in a high-throughput fashion. All the assays employ the same core strategy, whereby a hairpin loop oligonucleotide substrate is covalently bound by one end to wells of 96-well plates (Supplementary Figure S1). Each oligonucleotide complex carries a single internal lesion within the double-stranded region of the hairpin loop that is representative of a lesion or intermediate in the BER pathway. A fluorescein moiety is incorporated at the opposite end of the oligonucleotide structure to that bound to the wells of the plate. Purified recombinant DNA repair enzymes or repair enzymes present in nuclear extracts act on the substrate, either creating or removing an SSB or an AP site in the complex. During the subsequent denaturation step, the end of the oligonucleotide carrying fluorescein will be lost from the wells of the plate if the oligonucleotide hairpin structure contains an SSB. If alkaline denaturation is used, AP sites are converted to SSB and the same effect is achieved. Consequently, enzyme activity is determined by quantifying the amount of fluorescein retained in (or eluted from) the wells as a result of the denaturation step.

These new assays have a number of key advantages; they are sensitive, high throughput, fully quantitative, cost-effective, non-radioactive and can quantitate each individual step of BER. In addition to step-specific analyses, we demonstrate that the assay format can also be used to quantify overall repair across multiple steps of BER. Therefore, we propose that our assays are uniquely suited for investigations of DNA repair capacity as a potential health-related biomarker.

## MATERIALS AND METHODS

### Reagents

All chemicals were purchased from Sigma-Aldrich (Dorset, UK), unless otherwise specified. Nunc^®^ Immobiliser™ amino 96-well plates and Halt™ protease inhibitor cocktail were purchased from ThermoFisher Scientific (Hemel Hemstead, UK). Synthetic oligonucleotides were purchased from Integrated DNA Technologies (Leuven, Belgium) and Sigma-Aldrich, and were purified by high-performance liquid chromatography. Recombinant T4 DNA ligase, 3′ → 5′ exonuclease minus Klenow fragment of *Escherichia coli* DNA polymerase I (exo^−^ Klenow), deoxynucleotide triphosphates (dNTPs) and herring sperm DNA were purchased from Promega (Southampton, UK). Recombinant human POLB (19), purified by his-tag chromatography using an imidazole gradient elution (20), was a generous gift from Dr Jason Parsons (University of Liverpool, UK). All other recombinant DNA repair enzymes and uracil glycosylase inhibitor (UGI) were purchased from New England Biolabs (Hitchin, UK). L189 mammalian DNA ligase inhibitor was purchased from Bio-Techne (Abingdon, UK). Cell lines were purchased from ATCC (Middlesex, UK) and cell culture consumables were purchased from Lonza (Slough, UK). SV40 T-antigen transformed wild-type MEFs were a generous gift from Prof. Leona D. Samson (MIT, USA).

### Cell culture

The human HepG2 hepatocellular carcinoma (21), Caco-2 colorectal adenocarcinoma (22), ARPE-19 retinal pigmented epithelium and both wild-type and *PolB* null 88TAg immortalized mouse embryonic fibroblast (MEF) (23) derived cell lines were cultured in Dulbecco’s Modified Eagle’s Medium (DMEM) containing 25 mM glucose, and supplemented with 10% v/v fetal bovine serum, 100 units/ml penicillin, 100 units/ml streptomycin, 2 mM l-glutamine and 1× non-essential amino acids. *AAG* knockout ARPE-19 cells were generated using an *AAG*-specific CRISPR/Cas9 Knockout (KO) Plasmid system obtained from Santa Cruz Biotechnology (Dallas, TX, USA) following manufacturer’s protocols. Cells were incubated in a humidified atmosphere at 37°C with 5% carbon dioxide and harvested at approximately 80% confluence. Harvested cell pellets were stored at −80°C prior to nuclear extraction and protein quantification.

### Immunoblotting

Cells were lysed with M-PER Mammalian Protein Extraction Reagent ThermoFisher (Hemel Hemstead, UK), supplemented with 1× Halt protease inhibitor cocktail ThermoFisher (Hemel Hemstead, UK). Protein concentration was determined using the BCA assay (Pierce). Total protein lysates (20 μg) were separated under denaturing conditions in Any kD Mini-Protean TGX (BioRad, USA) gels. Proteins were then transferred onto polyvinylidene difluoride membranes (BioRad, USA). Membranes were blocked in 1% non-fat milk and incubated overnight at 4°C with anti-β-actin (1:7000 dilution; ab52614, Abcam) and anti-AAG (1:500 dilution; HPA006531, Sigma-Aldrich) antibodies. After primary antibody incubation, membranes were washed and then incubated with the secondary antibodies IRDye 680RD green goat anti-rabbit IgG and IRDye 800CW red goat anti-mouse IgM (LI-COR Biosciences, Lincoln, USA) at 1:10 000 for 1 h at room temperature. Proteins were detected using the Odyssey CLx IR imaging system (LI-COR Biosciences, Lincoln, USA).

### Peripheral blood mononuclear cell isolation

Ethical permission for this work was obtained from the University of Surrey Ethics Committee prior to the start of the study. All volunteers provided written informed consent and all aspects of the work were performed in accordance with the principle of the Declaration of Helsinki. Each volunteer provided a single blood sample of 20 to 50 ml obtained by venepuncture of an antecubital vein. The blood was collected into vacutainers containing potassium ethylenediaminetetraacetic acid (EDTA) anticoagulant. Peripheral blood mononuclear cells (PBMCs) were isolated using the Optiprep™ method as described previously (24). The PBMC pellets were snap frozen and stored at −80°C for subsequent extraction.

### Nuclear extraction and protein quantification of samples

Nuclear extraction was carried out on each cell pellet using a nuclear and cytoplasmic extraction reagents (NE-PER) kit (Thermo Fisher Scientific, Hemel Hemstead, UK) according to the manufacturer’s instructions, with the addition of 1× Halt™ protease inhibitor cocktail to the extraction reagents. The protein concentration of each nuclear extract was quantified using a microBCA kit (ThermoFisher Scientific, Hemel Hemstead, UK) according to manufacturer’s instructions. Nuclear extracts were frozen in aliquots at −80°C before thawing for DNA repair enzyme activity assays.

### Oligonucleotide-based assay for quantifying DNA repair enzyme activity

#### Substrate preparation

For all experiments, the initial oligonucleotide (URA03, HX02, AP02, Contr01, GAP04, LIG03 or HX02; sequence information provided in Table 1) was diluted from 10 μM stock solutions into freshly prepared 0.1 M bicarbonate buffer (pH 9.6) to a final concentration of 0.5 nM, and 100 μl of this solution was incubated in the wells of Nunc^®^ Immobiliser™ amino 96-well plates (Thermo Fisher Scientific, Hemel Hemstead, UK) overnight at 4°C. Plates were then washed with phosphate-buffered saline containing 0.1% v/v Tween-20 (PBST) and 100 μl of a 0.5 nM solution of the appropriate complementary oligonucleotide (Loop01Aflc or Loop01Gflc for URA03; Loop01Aflc, Loop01Cflc, Loop01Gflc or Loop01Tflc; Loop01A for AP02, HX02 or Contr01; Loop02 for LIG03 or GAP04; sequence information provided in Table 1) was added following dilution in hybridization buffer (6× saline sodium citrate (SSC) buffer, 5 mM EDTA, 0.1% v/v Tween-20). Each plate was then sealed and heated to 95°C for 10 min in a hybridization oven, followed by gradual cooling to 80°C, maintenance at 80°C for 10 min, and then further gradual cooling to 21°C.

**Table 1.** Sequences of oligonucleotides used in experiments

Name	Sequence (5′→3′)	Internal lesion^a^	Substrate for:
URA03	(P)CACGAA(U)CAACTCAGCAACTCCtt(NH_2_)^b,c,d,e^	Uracil (U)	UDG
HX02	(P)CACGAA(X)CAACTCAGCAACTCCtt(NH_2_)^f^	Hypoxanthine (X)	AAG
AP02	(P)CACGAA(B)CAACTCAGCAACTCCtt(NH_2_)^g^	Tetrahydrofuran (B)	AP incision
Contr01	(P)CACGAATCAACTCAGCAACTCCtt(NH_2_)	n/a	n/a
GAP04	(P)CAACTCAGCAACTCCtt(NH_2_)	Single nucleotide gap	DNA polymerase
LIG03	(P)TCAACTCAGCAACTCCtt(NH_2_)	Ligatable SSB	DNA ligase
Loop01Aflc	(flc)ttGGAGTTGCTGAGTTGATTCGTGAGCACCAACCGGTGCT^h^	n/a	n/a
Loop01Aird	(ird)ttGGAGTTGCTGAGTTGATTCGTGAGCACCAACCGGTGCT^i^	n/a	n/a
Loop01Cflc	(flc)ttGGAGTTGCTGAGTTGCTTCGTGAGCACCAACCGGTGCT	n/a	n/a
Loop01Gflc	(flc)ttGGAGTTGCTGAGTTGGTTCGTGAGCACCAACCGGTGCT	n/a	n/a
Loop01Tflc	(flc)ttGGAGTTGCTGAGTTGTTTCGTGAGCACCAACCGGTGCT	n/a	n/a
Loop02	(flc)ttGGAGTTGCTGAGTTGATTCGTGAGCACCAACCGGTGCTCACGAA	n/a	n/a

^a^Internal lesion present in oligonucleotide or in final substrate complex.

^b^P indicates phosphorylation.

^c^NH_2_ indicates amino modifier group.

^d^U indicates uracil.

^e^lower case letters indicate nucleotides linked via phosphorothiate bonds.

^f^X indicates hypoxanthine.

^g^B indicates tetrahydrofuran.

^h^flc indicates fluorescein.

^i^ird indicates IRDye®.

#### Uracil DNA glycosylase activity assay

To measure UDG activity, plates containing URA03 annealed to Loop01Aflc or Loop01Gflc were treated with 0.05U T4 DNA ligase in 100 μl of T4 DNA ligase buffer (30 mM Tris–HCl, pH 7.8, 30 mM NaCl, 10 mM MgCl_2_, 10 mM dithiothreitol (DTT), 1 mM ATP) for 1 h at 37°C. The plates were then heated to 65°C for a further 15 min and the hot liquid immediately decanted from the wells. Fresh DNA ligase buffer (100 μl) was added to each well and the plate heated to 65°C for a further 15 min, the liquid immediately decanted and the wells washed three times with PBST. This process produces a hairpin loop structure containing a single uracil lesion in a U:A base pair (with Loop01Aflc) or U:G base mismatch (with Loop01Gflc) within the double-stranded region. For the assays, the substrate was then incubated for 1 h at 37°C with varying concentrations of recombinant UDG or appropriate concentrations of nuclear extract sample, both in DNA repair buffer (20 mM Tris–HCl, pH 7.8, 100 mM NaCl, 0.4 mM EDTA, 3.4% v/v glycerol, 0.4 mM DTT, 2 mM MgCl_2_, 1 μg/ml herring sperm DNA). Following the repair incubation, the contents of the wells were decanted and the wells washed three times with PBST. A volume of 150 μl of alkaline buffer (0.1× SSC, 0.1 M NaOH) was added to the wells and the contents of the plate heated to 95°C for 10 min. The liquid was then decanted from the wells and the alkaline denaturation step repeated once more prior to three PBST washes. For the analysis of UDG inhibition by UGI, repair reactions were performed as described above using a fixed amount of either recombinant UDG or HepG2 nuclear extract in the presence of varying amounts of UGI. The amount of UDG or nuclear extract used for the inhibition assays was selected to achieve UDG activity in the wells toward the high activity end of the near linear portion of the UDG standard curve.

#### AP site incision activity assay

To measure AP site incision activity, substrate was prepared as for the UDG assay except that oligonucleotide AP02, containing an internal tetrahydrofuran (THF) group acting as an AP site analog, was bound to the plate and then hybridized and ligated to Loop01Aflc. This substrate was incubated with different concentrations of recombinant APE1 enzyme or nuclear extract samples in DNA repair buffer for 1 h at 37°C before denaturation as above except that neutral buffer (0.1× SSC buffer containing 0.1% w/v sodium dodecyl sulphate) was used in place of the alkaline buffer. To determine whether AP site incision activity was affected by the nucleotide opposite the THF, oligonucleotide Loop01Aflc was replaced with Loop01Cflc, Loop01Gflc or Loop01Tflc in some assays. To assess the effects of the APE1 inhibitor methoxyamine, immobilized substrate containing a single AP site was prepared by incubation of the UDG assay oligonucleotide complex with excess recombinant UDG (0.2 U/well) for 1 h at 37°C. This substrate was then incubated for 30 min with varying concentrations of methoxamine in PBS prior to performing the AP site incision assay as described above.

#### DNA polymerase activity assay

The substrate for the DNA polymerase assay was prepared by coupling oligonucleotide GAP04 to the plate and then hybridizing oligonucleotide Loop02 to it, to produce a double-stranded substrate containing a hairpin loop and a single nucleotide gap. DNA polymerase activity was determined by incubating the substrate with either various concentrations of recombinant exo^−^ Klenow or nuclear extract sample in the DNA repair buffer to which was added 2 mM ATP, 30 μM of each of dATP, dCTP, dGTP and dTTP plus an excess (0.05 U/100 μl) of T4 DNA ligase. Reactions were incubated for 1 h at 37°C. Denaturation was then achieved as described for the UDG assay.

#### DNA ligase activity assay

The substrate for the DNA ligase assay was prepared by coupling oligonucleotide LIG03 to the plate and then hybridizing oligonucleotide Loop02 to it to produce a double-stranded substrate containing a hairpin loop with a ligatable nick in the sugar-phosphate backbone. DNA ligase activity was determined by incubating this substrate with varying concentrations of either recombinant T4 DNA ligase or nuclear extract sample in DNA repair buffer containing 2 mM ATP and a final concentration of 10 mM MgCl_2_ for 1 h at 37°C. Denaturation was then achieved as described for the UDG assay.

To investigate the effects of the mammalian DNA ligase specific inhibitor L189 (25), repair reactions were performed as described above using a fixed amount of either recombinant UDG or HepG2 nuclear extract in the presence of varying amounts of L189. A 10 mM stock solution of L189 in DMSO was used to prepare the serial dilutions (the highest final DMSO concentration was 1% v/v). A set of control reactions were set up containing DMSO at final concentrations equivalent to those in the reactions containing L189 to account for possible effects of the vehicle. The cell extracts were incubated for 10 min at 25°C with the varying concentrations of L189 or vehicle before being cooled on ice again and then added to the assay plate wells.

#### Total AP site and DNA polymerase/dRP lyase assays

The substrate for these assays was prepared by treatment of the immobilized ligated URA03/Loop01Aflc substrate with excess UDG (0.2U/well) for 1 h at 37°C, as described for the methoxyamine inhibition assays, to generate a substrate containing an AP site. To measure complete AP site repair activity, this substrate was incubated with varying concentrations of nuclear extract in DNA repair buffer containing 2 mM ATP and 30 μM of each of dATP, dCTP, dGTP and dTTP and a final concentration of 10 mM MgCl_2_. To determine DNA polymerase/dRP lyase activity, an excess (0.05 U/well) of T4 DNA ligase was also included in each reaction mix. Denaturation was then achieved as described for the UDG assay except for one set of control wells, to which no nuclear extract had been added during the repair incubation, which were subjected to neutral rather than alkaline denaturation.

#### Alkyl adenine DNA glycosylase assay

AAG activity was determined in the same way as the UDG assay except that in place of URA03 an oligonucleotide (HX02) containing a single hypoxanthine was coupled to the surface of the plate and the repair reactions were performed with either recombinant human AAG or nuclear extract in 20 mM Tris (pH 7.5), 100 mM KCl, 5 mM EDTA, 1 mM EGTA, 5 mM β-mercaptoethanol, 1μg/ml herring sperm DNA.

#### Colorimetric detection of fluorescein

After the final denaturation step of each assay, fluorescein retained in the wells of the plates was quantified by colorimetric detection. Each well was incubated with anti-fluorescein antibody conjugated with horseradish peroxidase (HRP) (Abcam ab6656, Cambridge, UK) diluted 10 000-fold in PBST/1% w/v bovine serum albumin for 1 h at 21°C. The liquid was decanted from the wells and the wells washed four times with PBST. TMB Microwell Peroxidase Substrate for HRP (KPL, Wembley, UK) was then added (100 μl/well) and plates incubated at 21°C for 30 min. Reactions were stopped by the addition of 100 μl 1 M phosphoric acid to each well. Absorbance was read at 450 nm using an Omega Fluostar microplate reader (BMG Labtech, Aylesbury, UK).

### Characterization of oligonucleotides eluted from the plates after repair incubation for the UDG and APE1 assays

Oligonucleotide substrates attached to the wells on Nunc Immobilizer plates were prepared for the UDG and AP site incision assays as described above except that the oligonucleotide concentrations used were increased from 0.5 to 25 nM and oligonucleotide Loop01Aird (carrying an IRDye® 800CW modification in place of the fluorescein at the 5′ end) was used in place of Loop01Aflc. For each of the two assays, all 96 wells on one plate were treated as replicates and incubated with nuclear extract (3 μg/well Caco-2 extract for the UDG assay or 50 ng/well HepG2 nuclear extract for the AP site incision assays, respectively) for 2 h at 37°C under the assay conditions described above. The material eluted during the subsequent denaturation step was collected and pooled for all the replicate wells. The eluted oligonucleotide solutions were concentrated at 4°C using Vivapin 15R centrifugal ultrafiltration units with a 2 kDa nominal molecular weight cutoff (Sartorius, Epsom, UK). The concentrated samples were extracted with phenol:chloroform:isoamyl alcohol (25:24:1) and the aqueous phase then diafiltered into 20 mM Tris–HCl, 1 mM DTT, 1 mM EDTA (pH 8) and further concentrated using Vivaspin 500 centrifugal ultrafiltration units with a 3 kDa nominal molecular weight cutoff. The oligonucleotide samples eluted from the plates, unligated Loop01Aird, Loop01Aird ligated in solution to URA03, the product obtained after incubation of the in-solution ligated URA03-Loop01Aird with the recombinant *E. coli* UDG, and oligonucleotide length standard ladder (Integrated DNA Technologies, Leuven, Belgium) were electrophoresed through 15% polyacrylamide-urea gels, stained with and visualized using a LI-COR Odyssey® CLx imaging system to visualize all IRDye® 800CW labeled oligonucleotides and a LI-COR Odyssey Fc imaging system (LI-COR Biotechnology, Cambridge, UK) to visualize the position of IRDye® 800CW labeled oligonucleotides in relation to Sybr Green stained oligonucleotide length standard ladder.

### Data analysis

Data are presented as mean ± SD of at least three technical replicates unless otherwise stated. For the DNA polymerase and DNA ligase assays, data were background corrected by subtraction of the mean value obtained for the zero recombinant enzyme controls. Background corrected absorbance values were normalized to the maximum absorbance value of the assay standard curve. Nuclear extract enzyme activities were determined by interpolation from the recombinant enzyme standard curve, fitted with *y* = (*Ae^−Bx^*) +*C* for UDG, APE1 and AAG datasets, or *y* =*A*(*1-e^−Bx^*) for DNA polymerase and DNA ligase datasets. The parameter *E*_0_.*K*_Cat_/*K*_M_ was determined from time course data for the recombinant repair enzymes UDG, APE1 and AAG as the parameter B from the non-linear regression analyses performed using the equations above and *K*_Cat_/*K*_M_ determined based on the individual enzyme concentrations (*E*_0_) calculated from specific activity data provided by the supplier. Lower limits of detection for the assays were calculated by interpolation of the activity corresponding to the mean − 2 × SD of the absorbance for the zero enzyme controls of the UDG and APE1 standard curves or mean + 2 × SD of the absorbance for the zero enzyme controls of the exo^−^ Klenow fragment and T4 DNA ligase standard curves. Intra- and inter-assay coefficients of variation (CV) were calculated based on apparent enzyme activities, interpolated from recombinant enzyme standard curves, for quality control nuclear extracts prepared from HepG2 or Caco-2 cells. For the inhibitor analyses, IC_50_ values were estimated using the log(inhibitor) versus response curve fitting function in GraphPad Prism 7.0, and the effects of UGI, L189 and methoxyamine on other DNA repair enzyme activities were investigated by two-tailed one-way analysis of variance. Unpaired two-tailed *t*-tests were used to compare the BER enzyme activities in wild-type versus *PolB* null MEF and HepG2 versus Caco-2 cell line nuclear extracts. Spearman correlation analysis was performed with two-tailed *P* values calculated to examine the association between enzyme activities at individual steps of BER in the PBMC nuclear extracts.

## RESULTS

### Synthetic oligonucleotides immobilized in microplate well can be used to measure the activity of individual enzymes in the base excision repair pathway

For each assay, a synthetic oligonucleotide complex bearing a single modified base or lesion representative of an intermediate in the BER pathway was attached to the surface of a microplate (Supplementary Figure S1). First, a single oligonucleotide was covalently attached, via an amino modification at its 3′ end, to the surface of wells in Nunc^®^ Immobiliser™ amino 96-well plates. A second oligonucleotide, complementary to the first in the region of its 5′ end, with a hairpin loop structure at its 3′ end and a fluorescein moiety at the 5′ terminus was then hybridized to the first. The oligonucleotides were designed such that there was a two nucleotide ‘spacer’, composed of nucleotides joined by phosphorothiate bonds, between the NH_2_ group and the first base of the double-stranded section of the oligonucleotide complex. In the case of the UDG, AP site incision, AAG and DNA ligase assays, the oligonucleotides used form an immobilized double-stranded hairpin loop complex with a ligatable single strand nick between the 5′ end of the first oligonucleotide bound to the plate and the 3′ end of the second hybridized to the first. For the DNA ligase assay, this formed the final assay substrate. For the UDG, AP site incision and AAG assays, the nick was ligated by the incubation with excess T4 DNA ligase to generate intact hairpin loop structures. Note that we found that coupling of pre-hybridized and ligated complex to the wells resulted in unacceptably high background signal whereas in-well hybridization and ligation following coupling of the first oligonucleotide to the wells overcame this issue.

The UDG substrate formed contains a single uracil with an adenine or guanine opposite on the complementary strand depending on whether oligonucleotide Loop01Aflc or Loop01Gflc was used. The AP site incision substrate formed contains a single AP site analog with an adenine, cytosine, guanine or thymine opposite on the complementary strand depending on whether oligonucleotide Loop01Aflc, Loop01Cflc, Loop01Gflc or Loop01Tflc was used. For the AAG assay, the substrate formed contains a single hypoxanthine:thymine base pair. In the case of the DNA polymerase assay, the structure was essentially identical to that used for the DNA ligase assay except that there was a single nucleotide gap between the 5′ end on the first oligonucleotide and the 3′ end of the second.

The hairpin loop structure was incorporated into the assay substrate design for two reasons. First, following the ligation of the two oligonucleotides used to generate the UDG, AP site incision and AAG substrates, it is important to remove any unligated complexes, which could confound assay results if ligases present in the nuclear extracts acted on these complexes during the repair incubation step. The differential between the melting temperature of the unligated complex and the ligated hairpin structure is greater than it would be for an equivalent linear double-stranded complex melting temperature, making removal of the unligated oligonucleotide without denaturing the ligated complex easier. Second, the loop structure reduces the number of free strand ends accessible to degradation by non-specific exonucleases that could be present in cell extracts. As an additional protection against exonucleases, phosphorothioate linkages were incorporated between the three terminal nucleotides at the 3′ end of the oligonucleotides attached to the well surface and at the 5′ ends of the oligonucleotides hybridized to those attached directly to the wells.

Preliminary experiments were performed using a range of oligonucleotide concentrations to determine the minimum amounts that would provide adequate signal. Direct fluorescence detection of the fluorescein retained in the wells required an initial oligonucleotide concentration of ≥5 nM, whereas robust colorimetric detection of the fluorescein retained in the wells based on the use of HRP-conjugated anti-fluorescein antibody could be achieved with much lower oligonucleotide concentrations (≤0.5 nM) (data not shown). Based on this observation, the colorimetric detection system was used routinely in subsequent experiments.

#### Uracil DNA glycosylase activity

Due to our particular research interest in the effects of uracil misincorporation resulting from aberrant one carbon metabolism, we started the validation work for this assay using an oligonucleotide substrate containing an U:A base pair before going on validate it also for substrate containing an U:G mismatch, representative of the lesion arising from cytosine deamination. In initial experiments, performed with a substrate containing an U:A base pair, UDG activity in HepG2 nuclear extract proved difficult to detect, while the activity of recombinant *E. coli* UDG was readily detected. In some, but not all, experiments it appeared that there was a small decrease in signal with increasing nuclear extract concentration at the lower end of the range tested (typically in the range 0–0.5 μg/well) but at higher extract concentrations the UDG activity appeared to be progressively inhibited (data not shown). This led us to hypothesize that other nuclear proteins present in the extract were binding to the DNA and blocking access of the UDG to the lesion site. In support of this hypothesis, we found addition of excess recombinant *E. coli* UDG after incubation with higher concentrations of nuclear extract failed to excise the uracil and create alkali labile AP sites. In an attempt to counteract possible inhibition by non-specific binding of other nuclear proteins to the assay substrate, herring sperm DNA was added to the repair incubations to act as competitor for DNA-binding proteins. The presence of herring sperm DNA in the reaction mix did not interfere with the action of recombinant UDG, whereas its inclusion dramatically increased the apparent UDG activity in the nuclear extract (Supplementary Figure S2). For some of the other assays described below, in which lower concentrations of nuclear extract (<0.5 μg/well) were used, addition of herring sperm was not strictly required. However, for consistency, herring sperm DNA was included in all assays.

**Figure 1.**
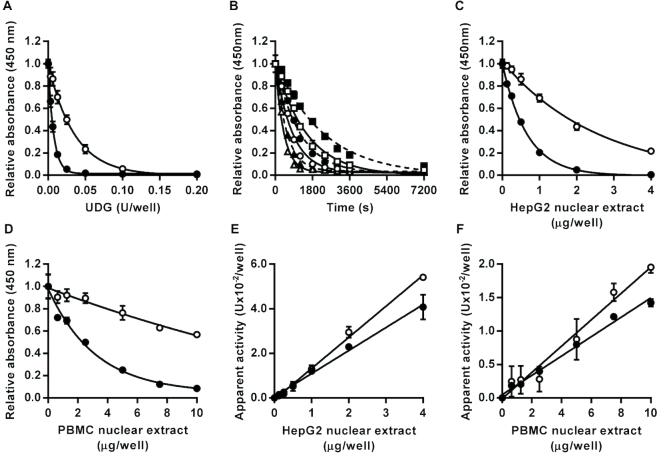
Effect of uracil DNA glycosylase activity on oligonucleotide substrates containing a single U:A base pair or U:G mismatch. Incubation with recombinant *Escherichia coli* uracil DNA glycosylase led to enzyme concentration dependent (**A**) and time-dependent (**B**) decreases in the amount of intact U:A (open symbols) and U:G (closed symbols) containing substrate retained in the wells after alkaline denaturation. For the time course analysis squares, circles and triangles indicate data generated with 0.1, 0.2 or 0.4 units/well, respectively, for the U:A containing substrate or with 0.0125, 0.025 or 0.05 units/well, respectively, for the U:G containing substrate. Incubation with HepG2 nuclear extract (**C**) or peripheral blood mononuclear cell pooled extract (**D**) also caused concentration-dependent decreases in the amount of intact U:A and U:G containing substrate retained in the wells. For both substrates, the apparent uracil DNA glycosylase activity was directly proportional to the concentration of HepG2 (**E**) and peripheral blood mononuclear cell (**F**) nuclear extract over the entire range tested (*r*^2^ = 0.99 in both cases). Data shown represent the mean ± SD of triplicate technical replicates except for time course data for which duplicates were analyzed.

When incubated with either the U:A base pair or U:G mismatch containing oligonucleotide substrates, recombinant *E. coli* UDG generated AP sites in the oligonucleotide substrate in a concentration-dependent manner as evidenced by the production of alkali-labile sites converted to strand breaks during the subsequent alkaline denaturation step, and so decreased retention of fluorescein-conjugated oligonucleotide detectable in the wells (Figure 1A). The enzyme acted approximately 5-fold more efficiently on the U:G containing substrate, consistent with the known substrate preferences of the family-1 UDGs (7). The parameter *K*_Cat_/*K*_M_, variously referred to as the specificity constant, the performance constant or the enzymatic proficiency (26), was determined by non-linear regression analysis of time course data generated for *E. coli* UDG acting on both substrates (Figure 1B). *K*_Cat_/*K*_M_ was estimated at 0.01 nM^−1^ s^−1^ for the substrate containing an U:G mismatch and as 0.002 nM^−1^ s^−1^ for the substrate containing an U:A base pair. Values of *K*_Cat_/*K*_M_ for *E. Coli* UDG we identified or derived from previously published work varied 500-fold from 0.0002 to 0.1 nM^−1^ s^−1^ (27–31), albeit with 4 of the 6 values falling in the range 0.01–0.06. Thus the values determined with our new assay format fall within, or close to, the central range of published estimates.

In the presence of herring sperm DNA, the expected concentration-dependent decrease in signal was observed with increasing amounts of HepG2 nuclear extract (Figure 1C) and PBMC nuclear extract (Figure 1D) for both the U:A and U:G containing substrate. As with the recombinant enzyme, both nuclear extracts excised uracil 4- to 6-fold more efficiently from the U:G containing substrate than the U:A containing substrate. For both the U:A and U:G substrates, the apparent absolute level of UDG activity in the pooled PBMC nuclear extract was approximately 7-fold lower than in the HepG2 nuclear extract. The apparent UDG activities in the HepG2 and PBMC nuclear extracts, determined by interpolation from the standard curve generated by the action of recombinant *E. coli* UDG on the relevant U:A or U:G containing substrate, were directly proportional to the amount of nuclear extract added over the entire range tested (0–4 μg/well for the HepG2 extract and 0–10 μg/well for the PBMC extract) (Figure 1E and F).

To confirm that the concentration-dependent signal reduction produced by the nuclear extract was specifically due to the excision of the uracil only, and not to non-specific degradation of the oligonucleotide substrate, a control substrate lacking any internal uracil or other DNA damage was incubated under the same conditions with recombinant *E. coli* UDG or HepG2 nuclear extract. Under these conditions, neither the recombinant UDG preparation nor the nuclear extract decreased the signal when incubated with the control substrate in contrast to the effect on uracil-containing substrate (Supplementary Figure S3). Moreover, gel electrophoretic analysis was used to characterize the material eluted during the denaturation step of the UDG assay. This demonstrated the eluate contained a unique detectable oligonucleotide that differed in size from Loop01Aird or Loop01Aird ligated to URA03 but co-migrated, as expected, with the fluorophore-labeled oligonucleotide produced when Loop01Aird hybridized and ligated to URA03 in solution was incubated with *E. coli* UDG and then heated under alkaline conditions to convert AP sites into SSB (Supplementary Figure S4).

We further evaluated the specificity of this assay by investigating the effects of the UDG specific inhibitor UGI from bacteriophage PBS2 (32). Inclusion of UGI in the repair incubations with the substrate containing an U:A pair caused concentration-dependent inhibition of UDG activity with an IC_50_ of 0.002 U/well for both the recombinant *E. coli* UDG and the UDG activity in HepG2 nuclear extract (Supplementary Figure S5A). In contrast, UGI at concentrations up to 2 U/well had no significant effect on the activity of other DNA repair enzymes in the HepG2 nuclear extract analyzed using the AP site incision, DNA polymerase or DNA ligase assays described below (Supplementary Figure S5B–D). Mammalian cells can express up to four known UDGs; UNG (with variant form UNG1 localized within mitochondria and UNG2 localized within the nucleus), TDG, SMUG1 and MBD4 (7). Of these, UGI only inhibits UNG1 and 2. Since different cell types have unique expression profiles of these different UDGs, we compared the effects of UGI on apparent UDG activity in HepG2 versus PBMC nuclear extracts. UGI, at 2U per reaction, reduced the apparent UDG activity by 85–100% for both cell extracts with both assay substrates (Supplementary Figure S5E) indicating that UNG2 is the predominant activity detected with this assay for both cell types.

#### AP site incision activity

**Figure 2.**
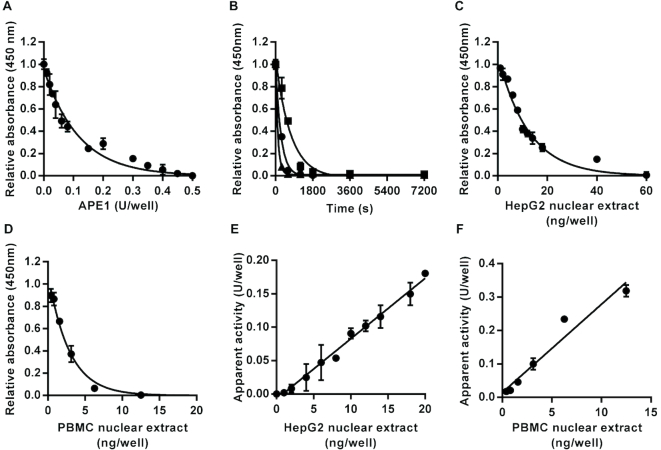
Effect of apurinic/apyrimidinic (AP) site incision activity on an oligonucleotide substrate containing a single tetrahydrofuran AP site analog. Incubation with recombinant human APE1 led to enzyme concentration-dependent (**A**) and time-dependent (**B**) decreases in the amount of intact substrate retained in the wells after neutral denaturation. For the time course analysis squares, circles and triangles indicate data generated with 0.1, 0.2 or 0.4 units/well, respectively. Incubation with HepG2 nuclear extract (**C**) or PBMC pooled nuclear extract (**D**) also caused concentration-dependent decreases in the amount of intact substrate retained in the wells. The apparent AP site incision activity was directly proportional to the concentration of nuclear extract over the range 0–0.02 μg/well for the HepG2 nuclear extract (*r*^2^ = 0.99) (**E**) and over the range 0–0.0125 μg/well for the peripheral blood mononuclear cell nuclear extract (*r*^2^ = 0.95) (**F**). Data shown represent the mean ± SD of triplicate technical replicates except for time course data for which duplicates were analyzed.

AP site incision activity was measured using a substrate containing a single THF, a synthetic AP site analog commonly used for research purposes (33,34). The THF was positioned at the same location in the oligonucleotide substrate as the uracil in the UDG substrate. Recombinant human APE1 acted on this substrate to produce strand nicks leading to concentration- and time-dependent decreases in the amount of intact THF-containing oligonucleotide bearing a 3′ fluorescein following neutral denaturation (Figure 2A and B). Based on the time course analysis, *K*_Cat_/*K*_M_ for the purified recombinant APE1 was estimated at 0.05 nM^−1^ s^−1^. Estimates of *K*_Cat_/*K*_M_ for APE1 derived from previous publications, we identified (35–44) varied approximately 50-fold from 0.026 (42) to 1.2 nM^−1^ s^−1^ (43). However, 5 of the 9 estimates were within the range 0.026–0.050 nM^−1^ s^−1^, in good agreement with the estimate of *K*_Cat_/*K*_M_, we obtained.

HepG2 and PBMC nuclear extracts also elicited concentration-dependent decreases in the amount of intact substrate retained in the wells (Figure 2C and D). AP site incision activity in the nuclear extracts was directly proportional to the amount of extract added in the range 0–0.02 μg/well for the HepG2 nuclear extract (Figure 2E) and 0–0.0125 μg/well for the PBMC nuclear extract (Figure 2F). Gel electrophoretic analysis of material eluted during the denaturation step confirmed that incubation of the substrate with HepG2 nuclear extract led to the release of a unique oligonucleotide that co-migrated with the oligonucleotide eluted during the denaturation step of UDG assay (Supplementary Figure S4).

We also evaluated the effect of the nucleotide opposite the THF lesion by comparing the AP site incision activities of recombinant APE1 and HepG2 nuclear extract acting on oligonucleotide substrates generated by hybridizing and ligating Loo01Aflc, Loop01Cflc, Loop01Gflc or Loop01Tflc to AP02 immobilized in the assay plate wells. Using this approach, we found that recombinant APE1 had essentially identical incision activity regardless of the nucleotide located opposite the THF (Supplementary Figure S6A). This observation is in line with the generalized function of APE1 to act at any AP site and with previous characterization of this enzyme (45). The HepG2 nuclear extract also exhibited similar AP site incision activity regardless of the type of nucleotide opposite the THF (Supplementary Figure S6B).

To our knowledge, there is currently no rigorously validated high specificity competitive inhibitor available for AP site incision activity of APE1, the major mammalian AP site endonuclease. However, methyoxyamine acts as a non-competitive inhibitor of APE1 by chemically modifying AP sites to a form that APE1 acts on much less efficiently (46). Therefore, we investigated the effects of methoxyamine pre-treatment of a substrate containing an AP site (generated by UDG treatment of the uracil containing substrate) on the AP site incision activity HepG2 nuclear extract. Pre-treatment with methoxyamine led to a concentration-dependent decrease in the degree of strand incision achieved when 4 ng/well HepG2 nuclear extract was used (Supplementary Figure S7A). The IC_50_ value estimated for this inhibitory action was 1.4 mM and no AP site incision activity was detectable when the substrate was pre-treated with methoxyamine concentrations of 5 mM or higher. However, incubation with 1 μg/well of the HepG2 nuclear extract in wells pre-treated with 25 mM methoxyamine led to a similar extent of substrate incision to that observed with 4 ng of HepG2 nuclear extract in the absence of methoxyamine pre-treatment (data not shown), consistent either with the reported ability of APE1 to act on methoxyamine modified AP sites at much reduced efficiency (47) or with the presence, at lower levels, of other enzymes capable of AP incision at methoxyamine modified AP sites in the extract.

Methoxyamine (25 nM) pre-treatment of the substrate for the UDG assay or the AP site incision assay substrate, containing the THF AP site analog, had no effect on the apparent UDG and AP site incision activity of HepG2 nuclear extract (Supplementary Figure S7B and C). On the other hand, methoxyamine (25 nM) pre-treatment of the substrates for the DNA polymerase and DNA ligase assays, described below, led to a moderate (approximately 25–50%) reduction in the enzyme activities detected in HepG2 nuclear extract using these two assays (Supplementary Figure S7D and E). These observations suggest that, at high concentrations, methoxyamine reacts weakly with substrates containing a single strand break to partially inhibit the strand ligation and perhaps the gap filling step but with intact oligonucleotide substrates it only reacts with natural abasic sites to create a form that is much more resistant to AP site incision.

#### DNA polymerase activity

**Figure 3.**
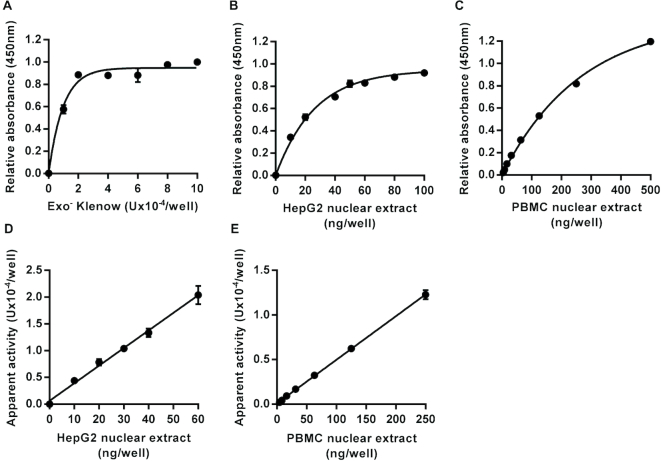
Assay for DNA polymerase repair activity. Incubation of an immobilized hairpin loop oligonucleotide substrate containing a single nucleotide gap on one strand within the double-stranded region with exonuclease minus Klenow fragment of *Escherichia coli* DNA polymerase I, in the presence of dNTPs, ATP and excess T4 DNA ligase, led to an increase in intact substrate retained in the microplate wells following neutral denaturation that was dependent on the concentration of the polymerase (**A**). Incubation with HepG2 nuclear extract (**B**) or peripheral blood mononuclear cell pooled nuclear extract (**C**) also caused concentration-dependent increases in the amount of intact substrate retained in the wells. The apparent DNA polymerase activity was directly proportional to the concentration of nuclear extract over the range 0–0.06 μg/well for the HepG2 nuclear extract (*r*^2^ = 0.98) (**D**) and over the range 0–0.25 μg/well for the peripheral blood mononuclear cell nuclear extract (*r*^2^ = 0.99) (**E**). Data shown represent the mean ± SD of triplicate technical replicates.

To determine the activity of DNA polymerases in nuclear extracts capable of working on single-nucleotide gaps with processed DNA backbone ends, the substrate prepared was identical to that used in the assays described above but with a single nucleotide gap at the same position in the sequence as the uracil and THF used for the UDG and AP site incision assays. This assay requires not only the action of DNA polymerase to fill the gap, but also DNA ligase to seal the single strand break, so that enzyme activity is detected as an increase in intact oligonucleotide carrying the 3′ fluorescein moiety following the repair reaction and subsequent denaturation step. Therefore, excess T4 DNA ligase was added to all reactions to ensure that the polymerase activity was rate limiting. A standard curve, constructed using serial dilutions of recombinant exo^−^ Klenow fragment, produced the expected concentration-dependent increase in signal (Figure 3A). Incubation of the substrate with HepG2 or PBMC nuclear extract also produced a concentration-dependent increase in signal (Figure 3B and C). The apparent DNA polymerase activity in the nuclear extracts, based on interpolation from the standard curve produced using exo^−^ Klenow, was directly proportional to the amount of extract added in the range 0–0.06 μg/well for HepG2 nuclear extract and 0–0.25 μg/well for pooled PBMC nuclear extract (Figure 3D and E). In the absence of dNTPs or ATP from the reaction mix, no repair activity was detected with the exo^−^ Klenow or with nuclear extracts (data not shown). Thus, omitting dNTPs and ATP from the reaction mixes for the DNA glycosylase and AP site incision assays avoids potential confounding of these assays by the action of DNA polymerases or DNA ligases in the nuclear extract.

#### DNA ligase activity

**Figure 4.**
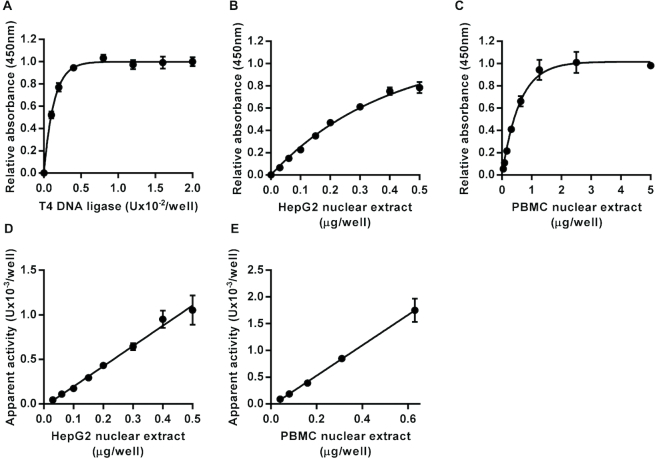
Assay for DNA ligase activity. Incubation of an immobilized hairpin loop oligonucleotide substrate containing a single ligatable nick in one strand within the double-stranded region with T4 DNA ligase, in the presence of ATP, led to an increase in intact substrate retained in the microplate wells following neutral denaturation that was dependent on the concentration of the polymerase (**A**). Incubation with HepG2 nuclear extract (**B**) and peripheral blood mononuclear cell pooled nuclear extract (**C**) also caused concentration-dependent increases in the amount of intact substrate retained in the wells. The apparent DNA ligase activity was directly proportional to the concentration of HepG2 (**D**) and PBMC (**E**) nuclear extract over the range 0–0.5 μg/well for the HepG2 nuclear extract (*r*^2^ = 0.99) and over the range 0–0.63 μg/well for the peripheral blood mononuclear cell nuclear extract (*r*^2^ = 0.99). Data shown represent the mean ± SD of triplicate technical replicates.

The substrate prepared for the DNA ligase assay was identical to that used in the DNA polymerase assay except that an additional nucleotide (T) was included at the 5′ end of the oligonucleotide attached via its 3′ end directly to the surface of the wells to generate an oligonucleotide complex containing a single ligatable nick Serial dilutions of recombinant T4 DNA ligase were used to generate the standard curve (Figure 4A). Again, incubation of the substrate with HepG2 or pooled PBMC nuclear extract produced a concentration-dependent increase in signal (Figure 4B and C). The apparent DNA ligase activity in the nuclear extracts, based on interpolation from the standard curve produced using T4 DNA ligase, was directly proportional to the amount of extract added in the range 0–0.5 μg/well for HepG2 nuclear extract, and 0–0.63 μg/well for pooled PBMC nuclear extract (Figure 4D and E). As with the DNA polymerase assays, no repair activity was detected with the DNA ligase assay when ATP was omitted from the repair reactions. Moreover, inclusion of L189, a specific inhibitor of mammalian DNA ligases I, III and IV (25), inhibited the DNA ligase activity in HepG2 nuclear extract in a concentration-dependent manner with an IC_50_ of 14 μM (Supplementary Figure S8A), very similar to the previously published IC_50_ values of 5 μM for both DNA ligases I and IV and 9 μM for DNA ligase III, determined using the individual purified enzymes (25). In contrast, T4 DNA ligase was much less sensitive to the presence of L189. At concentrations below 100 μM L189 had no significant effects of T4 DNA ligase activity and even at 100 and 200 μM, the highest concentrations tested, L189 only reduced activity to 89% and 84% of control values, respectively. L189 at a concentration of 200 μM had no significant effect on the BER activities of HepG2 nuclear extract measured using the UDG, AP site incision or DNA polymerase assays (Supplementary Figure S8B–D).

#### Assay sensitivity and reproducibility

The lower limits of detection for each of the single repair step assays described above were estimated based on the standard curves as explained in the methods section, and the minimum amount of HepG2 nuclear protein needed to detect activity was found using the lower limit of detection for each enzyme. The intra- and inter-assay coefficients of variation (CV) were determined by repeat analyses of nuclear extract quality control samples (Table 2). These analyses demonstrate that all four assays are highly sensitive and give good reproducibility, with intra-assay CVs generally <10% and inter-assay CVs generally <15%. Taken together, our results demonstrate that we have generated a sensitive and reproducible panel of microplate-based assays that are capable of measuring each enzymatically controlled step of the BER pathway.

**Table 2.** Sensitivity and reproducibility of DNA repair enzyme activity assays

Assay	Lower LoD (U/well)^a^	Amount of NE (ng/well)^b^	Intra-assay CV (%)^c^	Inter-assay CV (%)^d^
		1000	18 ± 3	18
UDG (U:A)^e^	0.009	2000	10 ± 2	10
		3000	8 ± 2	6
UDG (U:G)6	0.006	nd	nd	nd
		2	28 ± 11	8
AP site incision	0.005	4	14 ± 3	13
		8	12 ± 4	12
		12.5	12 ± 4	15
DNA polymerase	8 × 10^−7^	25	5 ± 1	9
		50	9 ± 3	6
		100	5 ± 1	6
DNA ligase	2 × 10^−6^	200	5 ± 2	10
		200	10 ± 4	20

^a^Lower limit of detection (LoD) calculated by interpolating enzyme activity for mean absorbance at 0U enzyme minus 2 × SD for uracil DNA glycosylase (UDG) and AP site incision assays, or mean absorbance at 0U enzyme plus 2 × SD for DNA polymerase and DNA ligase assays.

^b^Amounts of nuclear extract (NE) used for determinations of assay coefficients of variation (CV).

^c^Mean ± SD of intra-assay CV based on four individual analyses each of eight technical replicates.

^d^Inter-assay CV calculated from assays performed on four independent plates.

^e^Sensitivity and reproducibility of UDG assay measuring excision of uracil from a substrate containing an U:A base pair.

^f^Sensitivity of UDG assay measuring excision of uracil from a substrate containing an U:G mismatch. A HepG2 NE quality control sample was used for all assay CV determinations with the exception of UDG, for which a Caco-2 NE quality control sample was used.

**Figure 5.**
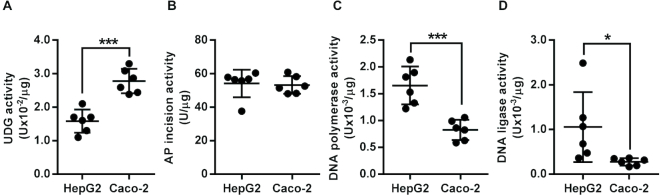
Within and between cell line variability in DNA repair enzyme activity of HepG2 and Caco-2 cells. (**A**) Uracil DNA glycosylase activity was higher in Caco-2 nuclear extract than HepG2 nuclear extract. (**B**) No significant differences were found in apurinic/apyrimidinic site incision activity between the two cell lines. (**C**) DNA polymerase activity was lower in Caco-2 nuclear extract than HepG2 nuclear extract. (**D**) DNA ligase activity was lower in Caco-2 nuclear extract than HepG2 nuclear extract. Bars indicate mean ± SD of six independent replicates. The symbols * and *** indicate significant differences between the enzyme activities of the cell lines at *P* < 0.05 and *P* < 0.001, respectively (based on two-tailed unpaired *t*-tests).

We next used these assays to compare BER enzyme activities in two different cells lines (HepG2 and Caco-2) using sets of nuclear extracts from each cell type that had been isolated over serial culture passages. The analyses demonstrated generally consistent levels of UDG, AP site incision, DNA polymerase and DNA ligase activity within each cell type but notable differences between the two cell lines (Figure 5). While the activity of AP site incision activity did not differ significantly between the two cell types, apparent UDG activity was 1.7-fold higher in Caco-2 cells than in HepG2 (*P* < 0.0005) whereas the DNA polymerase and DNA ligase activities were 2.0 and 3.8-fold lower, respectively, in Caco-2 cells than HepG2 (*P* < 0.0005 for DNA polymerase and *P* < 0.05 for DNA ligase). These data indicate that BER enzyme activities are relatively stable over time within each of these cells lines, that consistent activities can be obtained with the nuclear extraction protocol and that the assays can be used to distinguish cell-specific profiles of BER enzyme activities.

A key goal of this work was to develop assays suitable for use with samples collected from clinical studies. To evaluate this, we performed a pilot analysis of DNA repair enzyme activities in individual PBMC nuclear extracts derived from 13 apparently healthy volunteers (9 females and 4 males, aged 22–50). UDG, AP site incision, DNA polymerase and DNA ligase activities were quantified in PBMC nuclear extracts from each individual. Based on the observation of lower UDG activity detected when using the substrate containing an U:A base pair, the substrate containing an U:G base pair was used for the UDG assay of these samples.

**Figure 6.**
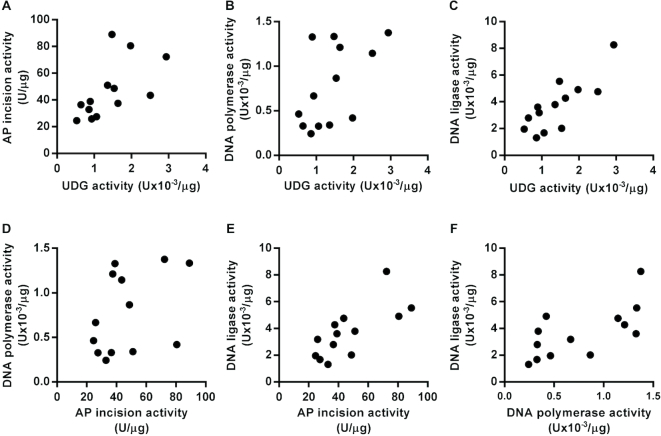
Base excision repair enzyme activity is correlated between some, but not all, enzymes of the pathway in nuclear extracts of peripheral blood mononuclear cells from different individuals. (**A**) Uracil DNA glycosylase (UDG) activity was positively correlated with apurinic/apyrimidinic (AP) site incision activity (*r* = 0.61, *P* < 0.05). (**B**) UDG activity was not significantly correlated with DNA polymerase activity (*r* = 0.58, *P* = 0.053). (**C**) UDG activity was positively correlated with DNA ligase activity (*r* = 0.81, *P* < 0.001). (**D**) AP site incision activity was not significantly correlated with DNA polymerase activity (*r* = 0.40, *P* = 0.177). (**E**) AP site incision activity was positively correlated with DNA ligase activity (*r* = 0.73, *P* < 0.01). (**F**) DNA polymerase activity was positively correlated with DNA ligase activity (*r* = 0.67, *P* < 0.05).

In line with previous reports, there was substantial inter-individual variation of the BER enzyme activities, ranging from 3.6-fold to 6.3-fold differences from maximum to minimum activities across the 13 volunteers for AP site incision and DNA ligase, respectively (15). Interestingly, there was also clear evidence of correlations between the activities of the different enzymes with statistically significant association between UDG and AP site incision activities (*r* = 0.703, *P* < 0.01), UDG and DNA ligase activities (*r* = 0.753, *P* < 0.005), AP site incision and DNA ligase activities (*r* = 0.786, *P* < 0.005) and DNA polymerase and DNA ligase activities (*r* = 0.725, *P* < 0.01) (Figure 6). This suggests that the activity of these different BER enzyme may be coordinated. However, these data are from a small pilot study, and analysis of samples from a much larger cohort is required to confirm these associations.

#### Total AP site repair and DNA polymerase/dRP lyase assays

In addition to use for analyzing repair enzyme activities at individual steps of BER, this assay format has the potential to be used to determine combined enzyme activity over multiple steps in BER. We tested this using the AP site as a starting point since this is a common stage in the repair of many lesions processed via BER (3). The substrate for these assays was generated by treating the U:A base pair containing UDG assay substrate with excess recombinant *E. coli* UDG within the wells of the plates to generate immobilized hairpin oligonucleotides containing a single AP site. This strategy was favored over use of the substrate for the AP site incision assay because while the THF moiety incorporated as an AP site analog is acted upon by APE1, the 5′ blocking group left behind cannot be excised by POLB (48). Therefore, we would predict that repair of the substrate containing the THF lesion could not proceed via short patch BER.

**Figure 7.**
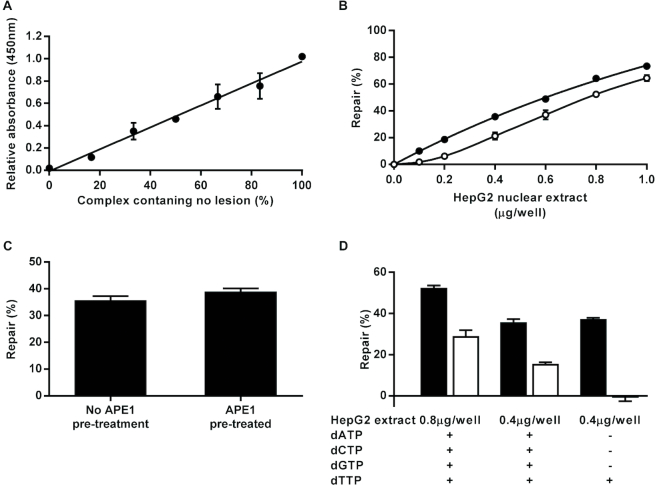
Complete repair of an apurinic/apyrimidinic (AP) site or a tetrahydrofuran AP site analog by HepG2 nuclear extract. The data presented in (**A**) demonstrate that color development observed in the assay plate wells is directly proportional to the amount of fluorescein modified oligonucleotide retained in the wells (*r*^2^ = 0.99). These data were generated by (i) coating the wells of a Nunc^®^ Immobilizer™ plate with 0.5 pmol/well of mixtures containing varying proportions of oligonucleotides URA03 (containing a single uracil lesion) and Contr01 (lesion free control), (ii) hybridizing and ligating a complementary oligonucleotide (Loop01Aflc) to the immobilized URA03/Contr01 mixtures to form intact hairpin loop structures covalently attached to the plate at the 3′ end with a fluorescein moiety at the 5′ end, (iii) incubating the immobilized substrates with excess *Escherichia coli* uracil DNA glycosylase to excise the uracil from those oligonucleotide carrying them to create AP sites, (iv) subjecting the substrate to hot alkaline denaturation to convert all AP sites into single strand breaks and remove any oligonucleotides released as a result and (v) detecting fluorescein retained in the wells using anti-fluorescein horseradish peroxidase conjugate and TMB substrate. Based on this observation, percentage repair was determined by linear interpolation from the absorbance values for 0 and 100% repair control wells. (**B**) Effects of varying concentrations of HepG2 nuclear extract on the extent of complete AP site repair in the absence (open circles) and presence (closed circles) of excess T4 DNA ligase. (**C**) Comparison of extent of complete repair by HepG2 nuclear extract of an oligonucleotide complex containing an AP site that had either been pre-treated or not with excess recombinant human AP endonuclease 1. (**D**) Comparison of the extent of complete repair of an oligonucleotide containing either a normal AP site (black bars) or the tetrahydrofuran AP site analog (white bars) by 800 or 400 ng/well of HepG2 nuclear extract in the presence of either 30 μM of all four deoxynucleotides (dATP, dCTP, dGTP and dTTP) or only 30 μM dTTP. Error bars indicate mean ± SD of triplicate technical replicates.

Complete repair of the AP site generated from the URA03/Loop01Aflc complex should lead to incorporation of a deoxythymidine at the lesion site and the resultant repaired complex will be resistant to alkaline denaturation. Since this is a multi-step repair process, a standard curve cannot be generated using serial dilutions of a single recombinant enzyme in the same way as for the assays described above. However, by mixing oligonucleotides URA03 and Contr01 in different ratios prior to coupling the oligonucleotide mixtures to the microplate wells, we were able to generate hairpin loop structures with Loop01Aflc that contained varying proportions of uracil lesions and were then able to demonstrate that the color developed following treatment of these with excess UDG and subsequent alkaline denaturation was directly proportional to the amount of substrate retained in the wells (Figure 7A). Thus, by including control wells in the complete AP site repair assay that were treated with no cell extract and then subjected either to alkaline or neutral denaturation we are able to determine the signal equivalent to zero and 100% repair, respectively, and from this interpolate percentage repair achieved with different amounts of nuclear extract.

With this assay format, a concentration-dependent increase in percentage repair of the AP site-containing substrate was observed using HepG2 nuclear extract ranging from 0 to 1 μg/well (Figure 7B). We hypothesized, given the very high levels of AP site incision activity determined in the cell extracts, that incision at the AP site would not be a rate-determining step in this assay. In support of this we found that the extent of repair completed was almost identical regardless of whether or not the AP site-containing substrate was treated with excess recombinant APE1 prior to repair incubation with 0.4 μg/well HepG2 nuclear extract (Figure 7C). Moreover, the extent of repair was essentially identical regardless of whether a mixture of all four dNTPs (30 μM each) was included in the repair incubation or 30 μM of only dTTP, suggesting that short patch repair predominated (Figure 7D). Given these observations, one or more of the three other steps (gap filling by a DNA polymerase, removal of the 5′ dRP group by a dRP lyase and strand ligation) must determine the overall percentage repair observed. Therefore, we produced a variant of this assay in which excess T4 DNA ligase was included in the repair reactions. Additional T4 DNA ligase was initially tested at concentrations of 0.005 and 0.05 U/reaction on the basis that these were sufficient to drive the reaction to completion over 1 h in the DNA ligase assay (Figure 4A). We observed almost identical enhancement of the extent of complete AP site repair with both concentrations (data not shown), confirming that these T4 DNA ligase concentrations were sufficient to ensure the maximal effect. A more detailed analysis using the higher of these two T4 DNA ligase concentrations showed that the extent of repair was increased by approximately 8–12% compared with the assay performed in the absence of added T4 DNA ligase, depending on the concentration of nuclear extract used (Figure 7B). We propose that this format provides an assay specific for the combined gap filling DNA polymerase and dRP lyase activities of extracts.

When a substrate containing the THF AP site analog was used in place of an AP site generated by the action of UDG on a uracil containing oligonucleotide complex, repair of the substrate was achieved by the nuclear extract when a mixture of the four dNTPs was included in the repair reaction although the extent of repair was lower, at approximately 55% of that observed when a substrate containing a normal AP site was used (Figure 7D). Moreover, unlike the repair of a substrate containing a normal AP site, inclusion of dTTP as the only deoxynucleotide in the repair mixture was not sufficient to support any detectable repair of the THF-containing substrate (Figure 7D). This indicates that, as predicted, short patch repair cannot be completed using the THF containing AP site analog but that it can be repaired via an alternative process, which is mostly likely long patch BER.

To investigate the specificity of the assays for DNA polymerase, combined DNA polymerase/dRP lyase and complete repair of the substrate containing a THF AP site analog, we compared the activity detected with each assay using nuclear extracts prepared from wild-type and *PolB* null MEFs. Activity detected with all three assays was substantially lower in the *PolB* null nuclear extracts than in the wild-type samples (Supplementary Figure S9A–C), whereas UDG activity was 2-fold higher in *PolB* null extracts than in WT extracts (Supplementary Figure S9D). AP site incision and DNA ligase activities of the wild-type and *PolB* null cells were very similar (Supplementary Figure S9E and F). For the DNA polymerase assay, there was a 45-fold difference between wild-type and *PolB* null extracts (*P* < 0.0001). For the combined DNA polymerase/dRP lyase assay, there was 56-fold difference (*P* < 0.0001). For the assay determining complete repair of the substrate containing a THF AP site analog, there was a 9-fold difference (*P* < 0.0001). However, for the last of these assays the repair activity determined with the highest concentration of *PolB* null nuclear extract tested (2 μg/well) remained below the limit of detection. Thus, while there is clearly a striking difference in the capacity of wild-type and *PolB* null MEF nuclear extracts to repair the THF AP site analog, the apparent fold difference in activity may not be as accurate as the fold differences determined with the other two assays.

Finally, we evaluated the capacity of purified recombinant human POLB to rectify the repair deficiencies of *PolB* null extract observed using each of these three assays. In each case, addition of POLB was sufficient to complement the extract activity such that repair similar to, or even exceeding, that of the wild-type MEF extract could be achieved (Supplementary Figure S9G–I).

#### Alkyl adenine DNA glycosylase activity assay

**Figure 8.**
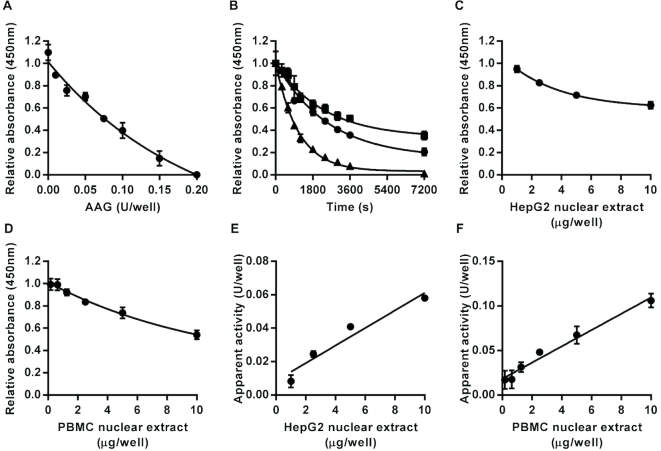
Assay for alkyladenine DNA glycosylase (AAG) activity. Incubation with recombinant human AAG led to enzyme concentration- (**A**) and time-dependent (**B**) decreases in the amount of intact oligonucleotide substrate containing an I:T base pair following alkaline denaturation. For the time course analysis squares, circles and triangles indicate data generated with 0.1, 0.2 or 0.4 units/well, respectively. Incubation with HepG2 nuclear extract (**C**) or peripheral blood mononuclear cell pooled extract (**D**) also caused concentration-dependent decreases in the amount of intact substrate retained in the wells. The apparent AAG activity was directly proportional to the sample concentration over the range 0–10 μg/well for HepG2 (**E**) and peripheral blood mononuclear cell (**F**) nuclear extracts (*r*^2^ = 0.94 in both cases). Data shown represent the mean ± SD of triplicate technical replicates except for time course data for which duplicates were analyzed.

Having demonstrated that the assay format can be used for each individual step in the BER of uracil lesions, we also evaluated the feasibility of extending the assay format to the repair of another base lesion by a different DNA glycosylase. The modified base hypoxanthine can be formed in DNA by the spontaneous deamination of adenine. This lesion is excised by AAG (49). Therefore, we prepared a hairpin loop oligonucleotide immobilized in assay plates that contained a single hypoxanthine (referred to as inosine (I) in the form of the deoxynucleoside), in an I:T base pair, as the substrate for the AAG assay. Incubation of this substrate with recombinant human AAG led to concentration- and time-dependent production of alkali-labile sites as evidenced by the reduction of fluorescein retained in the wells following the alkaline denaturation step (Figure 8A and B). From the time course analysis, *K*_Cat_/*K*_M_ was estimated at 2.8 × 10^−4^ nM^−1^ s^−1^. This compares with *K*_Cat_/*K*_M_ values for AAG derived from previous publications we identified (49–54), where the substrates used contained I:T base pairs, which varied more than 400-fold from 2.4 × 10^−5^ (54) to 0.01 nM^−1^ s^−1^ (50) but with 4 of the 6 estimates in the much narrower range of 2.4 × 10^−5^ to 6.4 × 10^−4^ nM^−1^.s^−1^. Thus, here again, the estimate we obtained for *K*_Cat_/*K*_M_ with the new assay format aligned well with the majority of previous estimates of this parameter.

Incubation of the I:T containing substrate with HepG2 or pooled PBMC nuclear extract also led to a concentration-dependent reduction in signal (Figure 8C and D). The apparent AAG activity in the nuclear extracts, based on interpolation from the standard curve produced recombinant AAG, was directly proportional to the amount of extract added in the range 0–10 μg /well (Figure 8E and F).

To evaluate the specificity of this assay for AAG, we compared the AAG expression and apparent AAG activity in nuclear extract from ARPE19 cells (wild-type for AAG) with that in three different clonal *AAG* knockout cell lines derived from the parental cell line. AAG protein was detected by western blot analysis in extract prepared from the original ARPE19 cells line but not in any of the three knockout cell lines (Supplementary Figure S10A). The apparent activity in each of the knockout cell lines was very low; well below the assay limit of detection whereas AAG activity was readily detectable in the cells expressing AAG (Supplementary Figure S10B) while UDG activities were similar across all the cell lines (Supplementary Figure S10C).

## DISCUSSION

### Development of a robust method for the measurement of BER enzyme activity

The work described here details the development and validation of a panel of assays for quantifying enzyme activity at each individual step in the BER pathway. We have demonstrated that these assays can be used to determine DNA repair enzyme activity not just with purified recombinant enzymes but also within the complex matrix of mammalian cell extracts, and that the quantities of extract required for the analysis are sufficiently small that enough material for detailed analysis can be obtained even where the amount of starting material is limited, such as with blood samples from human volunteers. Moreover, the reproducibility of the data generated is comparable with other microplate assays such as ELISAs.

When evaluating the estimates of *K*_Cat_/*K*_M_ obtained using the new assay format for the recombinant BER enzymes *E. coli* UDG, human APE1 and human AAG, we noted substantial variation in the range of published values. The scale of this variation has been commented on previously (55) and sources of the variation are likely to include the assay format, the reaction conditions, whether a single or double-stranded substrate was used, for double-stranded substrates the type of nucleotide opposite the lesion and the overall sequence context of the lesion (27,30,31,53,55). However, for all of three enzymes the majority of published values fall within considerably tighter ranges and the values we have derived are within, or very close to, the these estimates. This supports the concept that these DNA repair enzymes are able to act on the immobilised oligonucleotides in a similar fashion to oligonucleotide substrates in solution.

We also demonstrated the specificity of the new assays via a combination of different approaches. Using an oligonucleotide with no internal lesions, we confirmed that the nuclear extracts do not non-specifically degrade the immobilized oligonucleotide substrate but that they do act upon oligonucleotide substrates containing a single uracil or THF AP site analog to release a substrate fragment of the expected size following the denaturation step. The family-1 UDG inhibitor UGI was shown to inhibit the activity of both recombinant *E. coli* UDG and the UDG activity of HepG2 nuclear extract with a very similar IC_50_ but had no detectable effect on the HepG2 nuclear repair activities detected using any of the other single step specific repair assays. UNG is the mammalian representative of the family-1 UDGs and is expressed in two variant forms: UNG1 is localized to mitochondria and UNG2 is localized to nuclei (7,56). Thus our analysis suggests that the UDG assay detected primarily UNG2 in both the HepG2 and PBMC nuclear extracts. This is further supported by the observation that the recombinant *E. coli* UDG and the UDG activities present in the both nuclear extracts exhibited a similar moderate preference for substrate containing an U:G mismatch over substrate containing a U:A base pair (57). Furthermore UNG2 has been reported to be the predominant UDG found in the nuclei of proliferating mammalian cells but UNG2 expression has been found to be lower in non-proliferative cells (57) and in leukocytes and PBMC preparations (57,58). This aligns with our observation of substantially lower UDG activity in the PBMC nuclear extract compared with the HepG2 nuclear extract.

Methoxyamine pre-treatment of substrate containing an AP site dramatically reduced AP site incision by HepG2 nuclear extract with an IC_50_ of 1.4 mM. However, AP site incision was readily detectable when the concentration of HepG2 nuclear extract was increased by 200-fold above that normally required for the assay even when the substrate had been pre-treated with 25 mM methoxyamine. While this does not conclusively confirm that the predominant AP incision activity being detected using this assay with nuclear extracts is APE1, these observations are entirely consistent with that situation since APE1 is an abundant ubiquitously expressed enzyme that has been reported to account for nearly all of the AP site incision activity observed in cell extracts (59) and its activity on AP sites is dramatically reduced (approximately 330-fold), but not completely blocked, by methoxyamine modification of AP sites (47). We observed that pre-treatment of the substrate for the UDG assay or of the substrate for the AP site incision assay containing the THF AP site analog had no effect supporting the specificity of the reaction between methoxyamine and normal AP sites in DNA. However, as far as we are aware the observation that very high levels of methoxyamine weakly interacted with substrates containing a single strand nick to partially inhibit DNA ligation and/or gap filling is novel.

The specificity of the DNA ligase assay was demonstrated by the fact that the assays requires a substrate containing a single ligatable nick (the presence of a single nucleotide gap at the nick completely blocked ligation unless the appropriate dNTP was also included), that the reaction is ATP dependent and that the apparent DNA ligase activity detected in HepG2 nuclear extract was inhibited by L189 whereas L189 had only a marginal effect on the activity of T4 DNA ligase and no detectable activity on UDG, AP site incision or DNA polymerase activity in HepG2 nuclear extract. Based on the evidence presented, it is not possible to ascertain exactly which DNA ligase (I, III or IV) is primarily responsible for the activity detected. This could be elucidated by the use of other specific inhibitory compounds such as L67 and L87 (25) or through the use of cells deficient for each specific ligase.

The latter approach was demonstrated with the AAG assay, the specificity of which was confirmed by the absence of any detectable AAG activity in *AAG* null ARPE19 cells. Equally, comparison of BER activities in wild-type and *PolB* null MEF nuclear extracts demonstrated that the absence of POLB dramatically reduced the activity detected with the all three of the new assays that require DNA polymerase gap filling activity. Both wild-type and *PolB* null MEF cell lines grow and divide rapidly and the nuclear extracts should contain significant replicative DNA polymerase activity. Therefore, the very low levels of activity detected in the *PolB* null nuclear extracts when using the DNA polymerase and combined polymerase/dRP lyase assays suggests that both assays discriminate effectively between the BER and replicative machinery.

The observation that addition of purified recombinant POLB to the *PolB* null extracts rectified the repair deficiencies in the DNA polymerase and combined DNA polymerase/dRP lysase assays is exactly as expected. Moreover, POLB has been proposed to play a vital role in long patch repair to fill the first nucleotide gap before either other DNA polymerases take over or POLB continues the synthesis of the long patch in a so-called ‘hit and run’ mechanism involving the flap endonuclease FEN1 (60,61). This is consistent with the finding that *PolB* null MEF nuclear extracts have extremely low capacity to repair the oligonucleotide substrate containing a THF AP site analog while addition of POLB to these extracts corrects this deficiency.

The amounts of nuclear extract required for each assay provide an indication of the relative activities of the enzymes within the extracts performing each step in BER *in vitro*. This suggests that AP site incision is performed most efficiently and that the second most efficient step is the gap filling performed primarily by POLB. The DNA ligation step was completed with either about the same efficiency (by HepG2 nuclear extract) or somewhat slower (by PBMC nuclear extract) than the gap filling step and that the least efficient steps in short patch BER were the excision of uracil by UDGs and the removal of the dRP lyase group from the 5′ end of the DNA at the strand incision site. The observations are in line with previous studies. For example, a previous kinetic analysis of UDG and APE1 action using molecular beacon and radiolabeled cleavage assays indicated that APE1 activity exceeds UDG activity in immortalized human cell lines (62) and the removal of a dRP blocking group following APE1 processing, carried out predominantly by the dRP lyase activity of POLB (63), has been suggested to be rate limiting in repair of AP sites by BER (55). However, more detailed analyses are required to confirm whether the dRP lyase activity is always the rate limiting enzyme activity in different cell types and the assays described here could be a very valuable tool to help perform such detailed kinetic analyses.

### Relative strengths of the novel assay in comparison to existing methods

These newly developed assays add to the range of DNA repair analysis methods available, each of which has its own strengths and weaknesses. Probably, the most extensively used format of DNA repair assay to date also employs synthetic double-stranded oligonucleotides as the substrate; however, these are used in solution rather than being immobilized (64). Most commonly the oligonucleotide carrying the internal lesion is radiolabeled at its 3′ end, although more recent studies have reported success in replacing the radiolabel with a fluorophore (65,66). After treatment with the recombinant enzymes or cell extract, the oligonucleotides are resolved on a denaturing acrylamide gel, and the activity of the enzymes is determined by the appearance or disappearance of shorter oligonucleotide fragments representing repair intermediates. Such assays have played a critical role in elucidating the mechanisms of action of repair enzymes, their substrate preferences and overall repair processes (14). A key limitation of these assays is that they are relatively labor intensive and so have only moderate throughput capacity.

A more recent variant on this type of assay makes use of molecular beacon oligonucleotide probes in place of the classical linear double-stranded oligonucleotides as substrates (62,67,68). These assays have the important advantage that they can provide real-time kinetic readouts for the rate of repair. These can only be applied to the early steps in the BER pathway that produce AP sites or single strand breaks, which destabilize the hairpin loop structure and so reduce the quenching of the fluorophores attached to the adjacent free ends of the molecular beacon.

Another oligonucleotide-based assay, known as the DNA repair biochip, involves fixing oligonucleotides with specific damage sites to a microplate well in order to measure several different glycosylase activities in cell extract in one well, with the end point measured as fluorescence (69). The biochip represents the most similar assay to the one presented here, with key differences being that our assay format can also be used to measure polymerase, ligase and AP site repair, and is quantitative due to the use of a recombinant enzyme standard curve.

Among other DNA repair assays available, host cell reactivation techniques represent an alternative for determining DNA repair activity in live cells, whereby cells are transiently transfected with plasmid reporter constructs carrying defined DNA damage, and DNA repair activity is detected within cells as their ability to repair the plasmid damage thereby activating or inactivating expression of the reporter gene (70–72). Advantages of this technique include the ability to introduce highly defined DNA damage into the plasmids *in vitro* and sensitive detection of overall repair of these lesions in live cells. Plasmid DNA rapidly acquires nucleosomes once transfected into mammalian cells but there are still some questions about how representative repair DNA in the context of a constitutively expressed plasmid gene is of DNA repair across different regions of the mammalian genome (15). Moreover, while host cell reactivation assays are a sensitive tool for detecting differences in cellular DNA repair capacity, they are not necessarily able to elucidate the changes in specific individual repair activities responsible for observed differences in overall repair efficiency.

The assays described here share the same limitation as other oligonucleotide-based DNA repair assays, that the substrates do not accurately reflect the complex local 3D structures of chromatin or the overall spatial organization of the genome within the cell nucleus, both of which are likely to be important in determining DNA repair efficiency. They also provide endpoint, rather than real-time, readout. However, they also have a number of key advantages. These include that the lesion in the oligonucleotide substrate is defined precisely, that individual steps in the repair process can be analyzed both separately and in combination, and that the assay format provides high throughput (with the possibility of assay automation), high sensitivity and that we have demonstrated rigorously that they can provide fully quantitative and reproducible determination of specific DNA repair enzyme activities within the complex protein mix of cell extracts. Additionally, the assay format has the flexibility to be used to determine the activity of other DNA glycosylases beyond those reported here, and potentially to enzyme activities in other DNA repair pathways. An important practical advantage is that the assay described here is that they can be performed entirely with reagents that are readily available from commercial sources without requirement for any specialised equipment, other than a microplate reader. Additionally, we have found that the assay plates can be stored at −20°C with PBST in the wells for several weeks after most steps in the assay protocol without appreciable effects on the results. This further facilitates batch processing. The most time consuming step of the assay is setting up the repair incubations, which involves preparing dilutions of the recombinant enzyme standards, quality controls and each test sample prior to pipetting the reaction mixes into the wells of the assay plates. Having prepared appropriate batches of assay plates, we have found that a competent laboratory worker can set up and complete the repair incubations for four to five 96-well plates in a day. Taking into account the blanks, standards and quality control samples included on each plate, this means it is feasible to analyze approximately 100–200 test samples per day depending on whether each sample is assayed in technical triplicate or duplicate. Use of a robotic liquid handling system for the assay setup would further increase the throughput capacity.

From the assay design perspective, it is worth noting that fluorescein was included at the free 5′ end of the hairpin loop oligonucleotide substrates, on the basis that this provided two alternative quantification strategies: direct quantification of fluorescein eluted from, or retained in, the well by fluorimetry or indirect quantification based on the use of an anti-fluorescein antibody-HRP conjugate and colorimetric detection using a suitable substrate for the HRP. For some of the assays, acceptable standard curves were generated by fluorimetric detection of the fluorescein eluted following the final denaturation step while the retained fluorescence signal proved more difficult to detect and quantify, even when black plates were used instead of clear ones (data not shown). Overall, we found the colorimetric detection method to be more sensitive and reliable. We also have preliminary results (not shown) that indicate similar results can be obtained using colorimetric detection strategies with biotin or digoxygenin used in place of the fluorescein. It may be that use of other fluorophores could enhance the sensitivity of the direct fluorescence detection approach and, if so, this would further streamline the assays by removing two steps in the protocol.

## CONCLUSION

The measurement of the BER pathway in this step-wise manner has a wide range of future applications. DNA repair activity has begun to be investigated as a disease risk predictor, and a reduction in BER capacity has been associated with lung cancer incidence (16). This is of particular relevance as early detection of cancers, such as lung cancer, reduces mortality by up to 20% (73). As previously mentioned, the association between BER function and disease risk is far from clear, in part due to the limitations in the measurement of the pathway thus far. The novel assays described here could be used in large prospective studies in combination with other techniques such as genetic and epigenetic analyses, quantitative PCR and western blotting, to investigate the molecular basis for the observed inter-individual variation in BER enzyme activities and links between BER enzyme activity and cancer risk both on an enzyme-by-enzyme basis and on overall repair activity profiles. Another important aspect to consider is the connection between BER capacity and chemotherapy resistance, where increased BER can lead to DNA-damaging agent resistance, and in this context an inhibition in BER capacity may be desirable (74).

This new assay format would also be ideal for further studies of repair enzyme specificity in relation to the sequence context of different lesions and as a robust bioassay format for screening candidate DNA repair enzyme inhibitors. Furthermore, the assays would be suitable to generate detailed datasets for mathematical modeling of flux through the BER pathway. Experiments could then be performed to investigate the effect of altering the rate limiting step on the build-up of intermediates, and overall pathway completion. Using these approaches, the most effective targets for drug and lifestyle interventions to improve, or indeed reduce, BER capacity can be investigated.

The panel of assays presented here provides an exciting opportunity to further explore various aspects of BER, including disease prediction, modulation of the pathway and interaction of BER with disease states.

## Supplementary Material

gkz171_Supplemental_File
