# Supplementary material for: A panel of colorimetric assays to measure enzymatic activity in the base excision DNA repair pathway
Source: Nucleic Acids Res. 2019 Mar 14;47(11):e61. doi: 10.1093/nar/gkz171 (PMC6582407; doi:10.1093/nar/gkz171)
Supplement: gkz171_Supplemental_File [file gkz171_supplemental_file.pdf]

Supplementary data

A panel of colorimetric assays to measure enzymatic activity in the base excision DNA repair pathway

Eleanor Healing[1], Clara F Charlier[2], Lisiane B Meira[2] and Ruan M Elliott[1,*]

[1] Department of Nutritional Sciences, University of Surrey, Guildford, Surrey, GU2 7XH, UK
[2] Department of Clinical and Experimental Medicine, University of Surrey, Guildford, Surrey, GU2 7XH, UK

* To whom correspondence should be addressed. Tel: +44 (0)1483 683843; Fax: +44 (0)1483 686401; Email: r.m.elliott@surrey.ac.uk

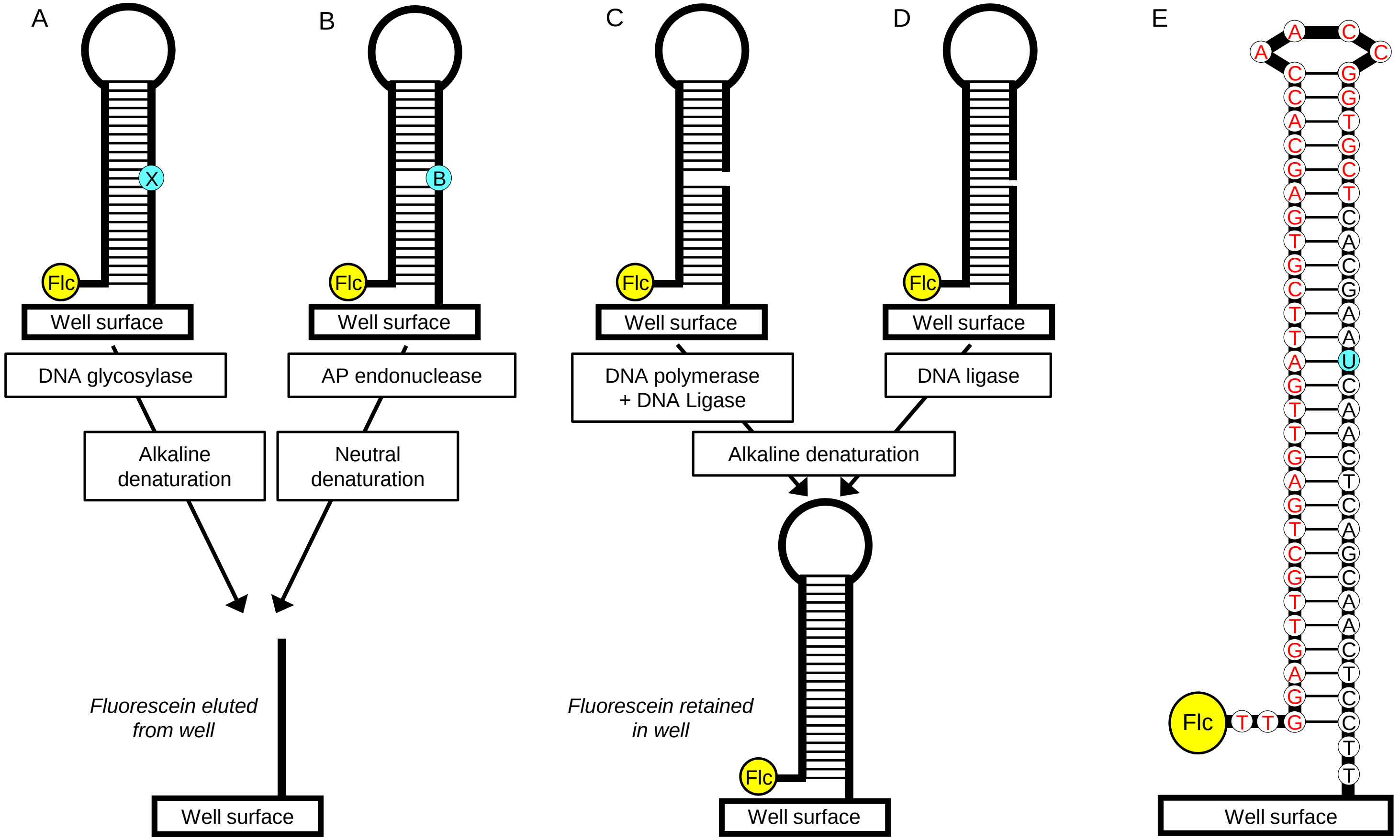

Supplementary figure 1. Assay schematic depicting the oligonucleotide substrates and strategies for measuring uracil or alkyladenine DNA glycosylases **(A)**, AP site incision **(B)**, DNA polymerase **(C)** or DNA ligase **(D)** activities. As an example, the full sequence and base pairing of the oligonucleotide substrate containing a U:A base pair for the uracil DNA glycosylase assay is shown **(E)**. White rectangles represent the assay microplate well surface. The nucleotide sequence in black text indicates the sequence of the oligonucleotide covalently bound to the surface. The nucleotide sequence in red text indicates the sequence of the oligonucleotide hybridized and, where appropriate, ligated to the surface-bound oligonucleotide. The thick black line connecting the nucleotides indicates the deoxyribose phosphate backbone. Thin lines between nucleotides indicate base pairing. Base lesions are highlighted in blue. X – base lesion substrate for a DNA glycosylase (uracil or inosine); B – tetrahydrofuran; Flc – 5' fluorescein).

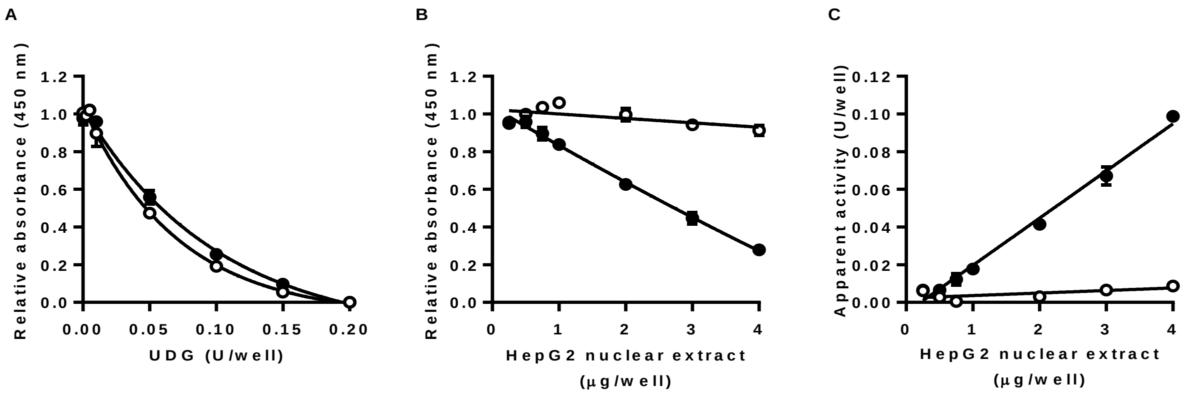

Supplementary figure 2. Effect of adding herring sperm DNA to the assay buffer on uracil DNA glycosylase activity of recombinant *E. coli* uracil DNA glycosylase and uracil DNA glycosylase activity in HepG2 nuclear extract. Open and closed circles indicate data generated in the absence or presence 0.2µg/well herring sperm DNA, respectively. Recombinant *E. coli* uracil DNA glycosylase activity was not affected by addition of herring sperm DNA to assay buffer **(A)**. The presence of herring sperm DNA in the assay buffer led to a much more marked concentration-dependent decrease in signal when oligonucleotide substrate was incubated with HepG2 nuclear extract **(B)** and consequently the apparent uracil DNA glycosylase activity in the HepG2 nuclear extract was greater in the presence of herring sperm DNA than in its absence **(C)**. Data shown represent the mean ± SD of 3 replicates.

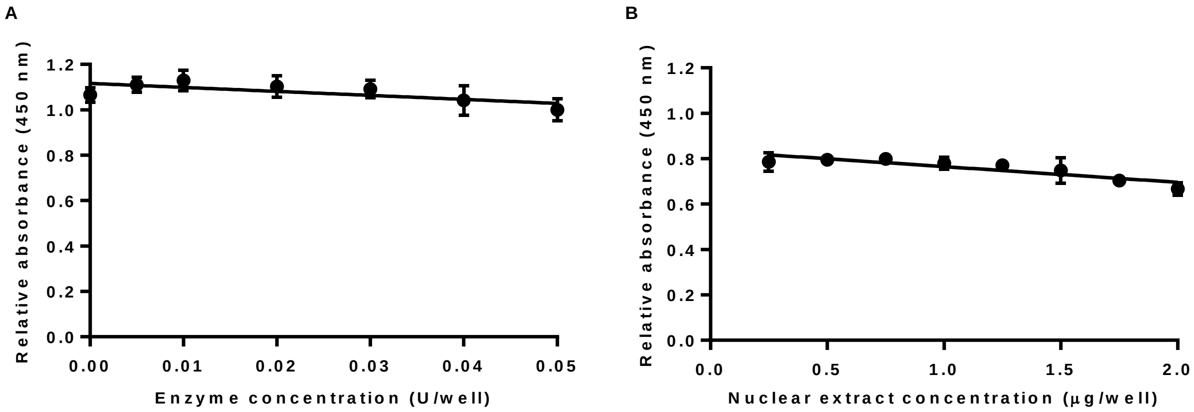

Supplementary figure 3. Effect of recombinant *E. coli* uracil DNA glycosylase and HepG2 nuclear extract on oligonucleotide substrate containing no lesions. Increasing concentrations of recombinant *E. coli* uracil DNA glycosylase **(A)** or HepG2 nuclear extract **(B)** had no apparent effect on substrate detectable on plate. Data shown as mean ± SD of 3 replicates.

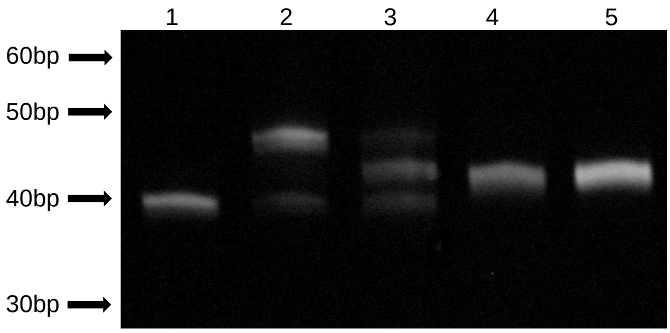

Supplementary figure 4. Characterization of oligonucleotides eluted from assay wells following denaturation for the uracil DNA glycosylase (UDG) and apurinic/apyrimidinic (AP) site incision assays. Oligonucleotide Loop01Aird (lane 1) migrated essentially as expected (apparent length based on gel motility ≈ 39 nucleotides versus actual length of 40 nucleotides plus the 5'fluorophore). Hybridization and ligation of URA03 to Loop01Aird gave rise to two products visible by gel analysis: some unligated Loop01A remained while a higher molecular weight band corresponding to the ligated complex was also visible (lane 2). The latter migrated somewhat faster than expected (apparent length based on gel motility ≈ 48 nucleotides compared with an expected length of 64 nucleotides plus the 5' fluorophore), most likely due to incomplete denaturation of the highly stable hairpin loop structure generated as a result of the ligation. A third band was evident when the ligated URA03-Loop01A complex was incubated in solution with *E. coli* UDG and then heated in alkaline solution to convert any AP sites to stand breaks (lane 3). This new product also migrated slightly faster than expected (apparent length based on distance migrated was approximately 43 nucleotides compared with an expected length of 46 nucleotides plus the fluorophore). The eluates obtained following incubation of the UDG and AP incision assay substrates immobilized in microplate wells with cell line nuclear extracts and subsequent alkaline denaturation (lanes 4 and 5, respectively) co-migrated with the fluorophore labelled oligonucleotide obtained following alkaline denaturation of the ligated URA03-Loop01Aird complex treated with *E. coli* UDG in solution.

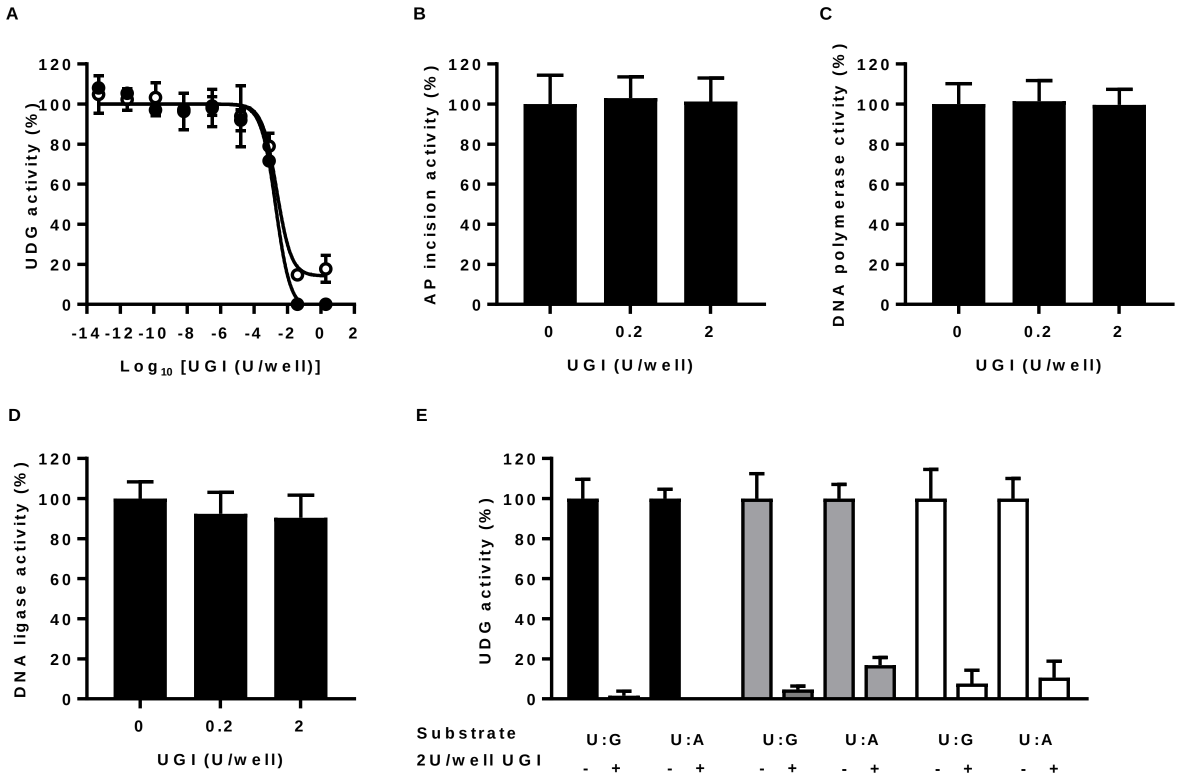

Supplementary figure 5. Effects of the uracil DNA glycosylase inhibitor UGI from bacteriophage PBS2 on base excision repair enzyme activities. UGI potently inhibited both *E. coli* uracil DNA glycosylase (closed circles) and the uracil DNA glycosylase activity in HepG2 nuclear extract (open circles) detected using a hairpin loop oligonucleotide substrate containing a U:A base pair within the double stranded region **(A)**. The IC_{50} concentration was estimated at 0.002U/reaction for both the *E. coli* recombinant enzyme and the HepG2 nuclear extract. In contrast, UGI at concentrations up to 2U/reaction had no detectable effect on the enzyme activities for apurinic/apyrimidinic site incision **(B)**, DNA polymerase **(C)** or DNA ligase activity **(D)** of HepG2 nuclear extract. The data presented in **(E)** demonstrate that UGI (2U/reaction) caused a similar degree of inhibition of *E. coli* uracil DNA glycosylase (black bars) or the uracil DNA glycosylase activity of nuclear extracts from both HepG2 cells (grey bars) and human peripheral blood mononuclear cells (white bars) regardless of whether substrate containing either a U:G mismatch or a U:A base pair was used. In **(A)** the error bars indicate mean±SD of 3 replicates. In **(B-D)** the bars and error bars indicate mean+SD for 9 replicates. In **(E)** the bars and error bars indicate mean+SD for 4 replicates.

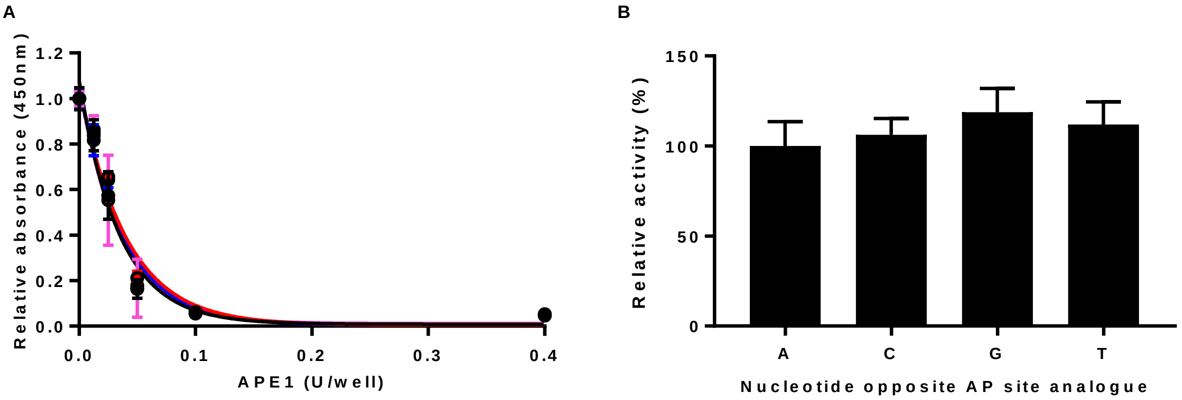

Supplementary figure 6. Effects of the nucleotide opposite a tetrahydrofuran apurinic/apyrimidinic (AP) site analogue on incision by recombinant human APE1 **(A)** or the AP site incision activity of HepG2 nuclear extract **(B)**. In **(A)** the black symbols and line indicate data generated with an A opposite the lesion; red symbols and line indicate data generated with a C opposite the lesion; blue symbols and line indicate data generated with a G opposite the lesion; pink symbols and line indicate data generated with a T opposite the lesion. The error bars indicate the mean±SD of 3 replicates. In **(B)** bars and error bars indicate mean+SD of 4 replicates.

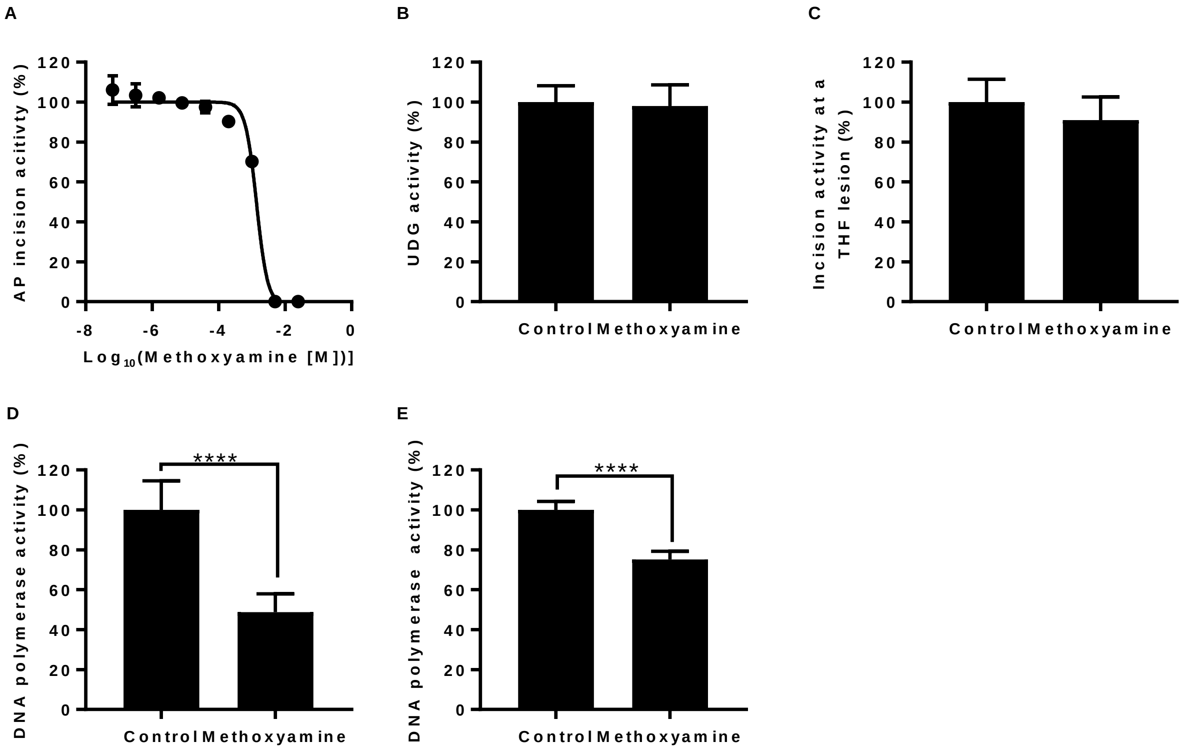

Supplementary figure 7. Effects of pre-treating immobilized hairpin loop oligonucleotide substrates with methoxyamine on base excision repair enzyme activities in HepG2 nuclear extract. Methoxyamine pre-treatment of an intact hairpin loop oligonucleotide substrate, which contained a single apyrimidinic/apyrimidinic (AP) site in the double stranded region, exerted a concentration-dependent inhibition of the HepG2 nuclear extract AP site incision activity with an apparent IC_{50} concentration of 1.4mM **(A)**. Conversely, high concentration (25mM) methoxyamine pre-treatment of intact hairpin loop oligonucleotide substrates containing either a single uracil **(B)** or a tetrahydrofuran analogue of an AP site **(C)** in the double stranded region had no detectable effect on HepG2 nuclear extract uracil DNA glycosylase or AP site incision activity. High concentration (25mM) methoxyamine pre-treatment of substrates containing a single stand break with a single nucleotide gap **(D)** or a ligatable single strand nick **(E)** in the double stranded region moderately reduced HepG2 nuclear extract DNA polymerase and DNA ligase activity. In **(A)** the error bars indicate mean±SD of 3 replicates. In **(B-E)** the bars and error bars indicate mean+SD for 6 replicates (B-E). The symbol **** indicates significant differences in the activities between control and methoxyamine treated substrates at $p<0.0001$ (based on 2-tailed unpaired t-tests).

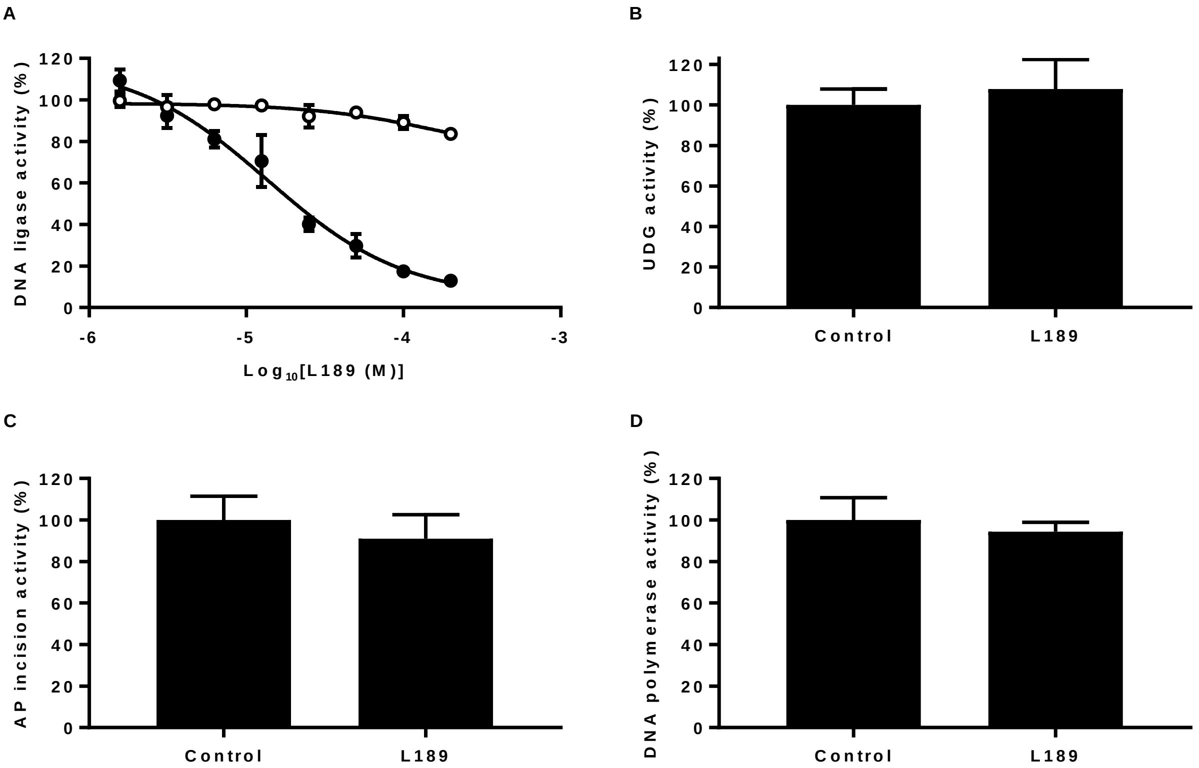

Supplementary figure 8. Effects of the mammalian DNA ligase inhibitor L189 on T4 DNA ligase and base excision repair enzyme activities in HepG2 nuclear extract. **(A)** L189 potently inhibited the DNA ligase activity in HepG2 nuclear extract (closed circles), with an estimated IC_{50} of 14µM, whereas it exerted a much weaker inhibitory effect on T4 DNA ligase (open circles) that was statistically significant only at the highest concentrations tested (100 and 200µM). L189 (at 200µM) had no detectable effects on HepG2 nuclear extract uracil DNA glycosylase **(B)**, apurinic/apyrimidinic site incision **(C)** or DNA polymerase **(D)** activities. In **(A)** the error bars indicate mean±SD of 3 replicates. In **(B-D)** the bars and error bars indicate mean+SD for 6 replicates.

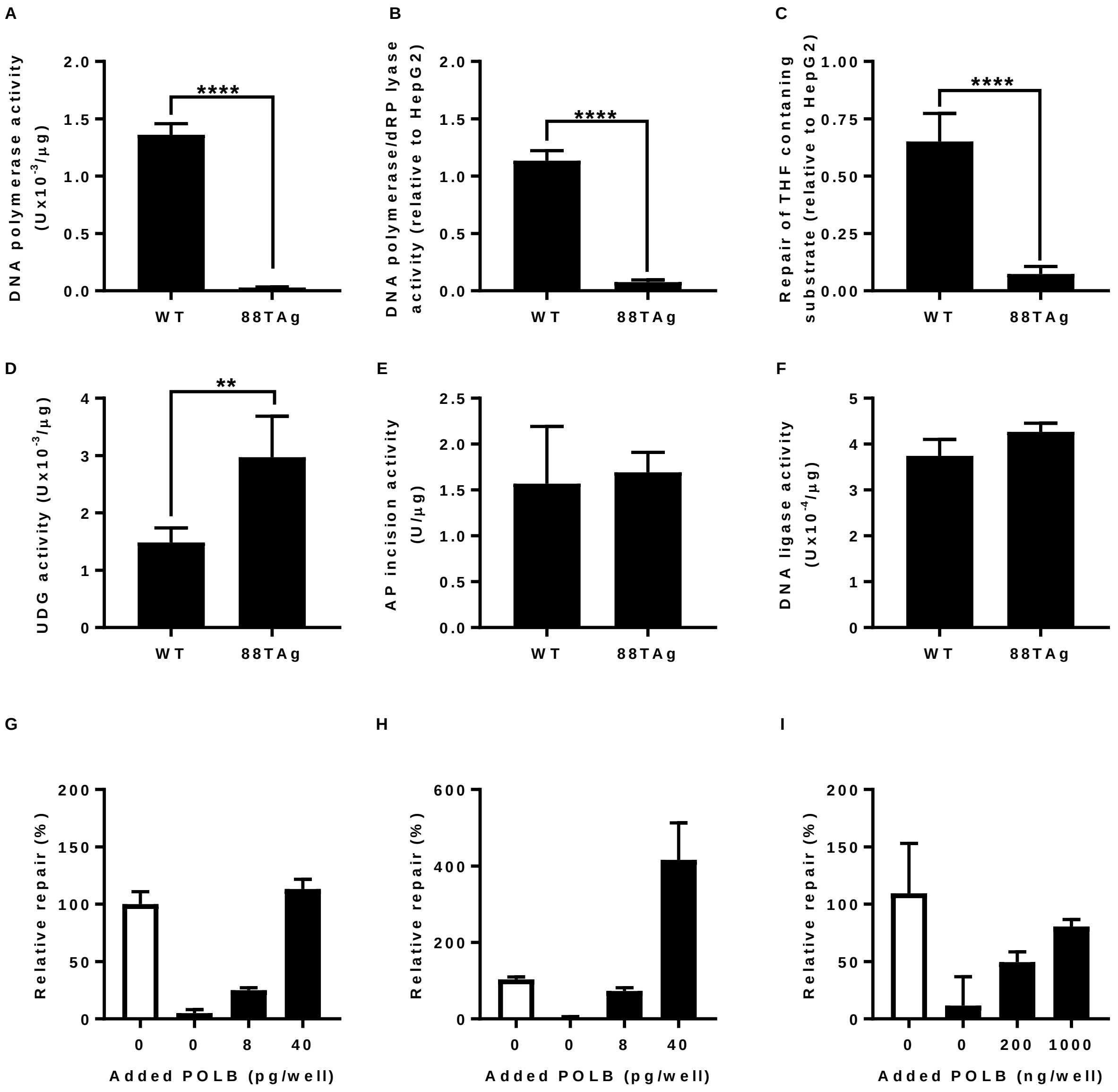

Supplementary figure 9. Comparison of base excision repair enzyme activities in nuclear extracts prepared from wild type (WT) and 88TAg (DNA polymerase β [*POLB*] null) mouse embryonic mouse fibroblasts. *POLB* null extracts exhibited markedly reduced activity compared to WT when analysed using the assays for DNA polymerase (**A**), combined DNA polymerase/deoxyribose phosphate lyase activity **(B)** or complete repair of substrate containing a tetrahydrofuran apurinic/apyrimidinic (AP) site analogue **(C)**. In contrast, uracil DNA glycosylase activity was higher in *POLB* null extracts than WT **(D)**, while AP site incision activity **(E)** and DNA ligase activity **(F)** did not differ significantly between the two cell types. Addition of purified recombinant human POLB enzyme to the *POLB* null extract rectified the repair deficiency observed with the assays for DNA polymerase activity (using 100ng/reaction of nuclear extract) **(G)**, combined DNA polymerase/deoxyribose phosphate lyase activity (using 100ng/reaction of nuclear extract) **(H)** or complete repair of substrate containing a tetrahydrofuran apurinic/apyrimidinic site analogue (using 2.5μg/reaction of nuclear extract) **(I)**. Bars and error bars indicate mean+SD for 4 independent replicates **(A-F)** or 6 technical replicates **(G-I)**. The symbols ** and **** indicate significant differences in the activities between cell types at $p<0.01$ and 0.0001, respectively (based on 2-tailed unpaired t-tests).

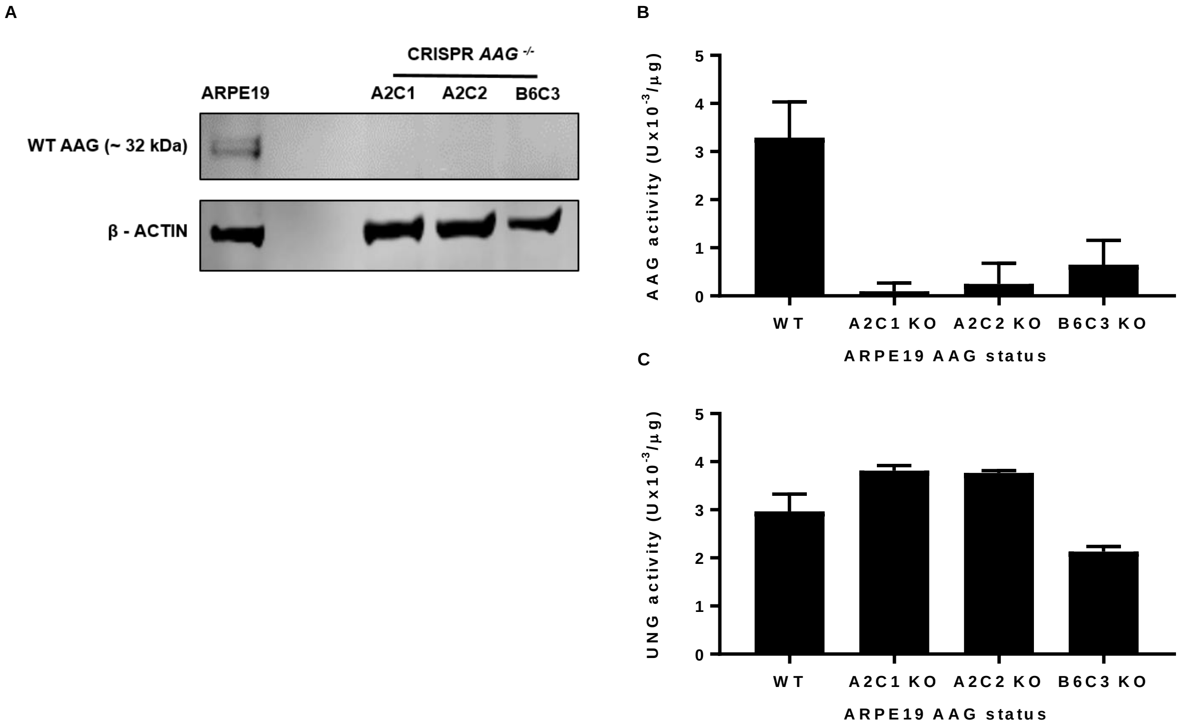

Supplementary figure 10. Comparison of base excision repair enzyme activities in nuclear extracts prepared from ARPE19 wild type (WT) and *AAG*$^{-/-}$ cell lines prepared using an AAG-specific CRISPR/Cas9 knockout (KO) plasmid system. Western blot analysis demonstrated the presence of detectable AAG protein in the WT but not in in *AAG*$^{-/-}$ ARPE19 cell extracts **(A)**. AAG activity was markedly lower in *AAG*$^{-/-}$ARPE19 cells compared to WT **(B)** whereas UDG activity was similar in *AAG*$^{-/-}$ARPE19 cell nuclear extracts compared to the activity in WT nuclear extract **(C)**. Bars and error bars indicate mean+SD for 3 replicates.
